# Timed Action of IL-27 Protects from Immunopathology while Preserving Defense in Influenza

**DOI:** 10.1371/journal.ppat.1004110

**Published:** 2014-05-08

**Authors:** Francesca Diane M. Liu, Elisabeth E. Kenngott, Micha F. Schröter, Anja Kühl, Silke Jennrich, Ralf Watzlawick, Ute Hoffmann, Thorsten Wolff, Stephen Norley, Alexander Scheffold, Jason S. Stumhofer, Christiaan J. M. Saris, Jan M. Schwab, Christopher A. Hunter, Gudrun F. Debes, Alf Hamann

**Affiliations:** 1 Deutsches Rheuma-Forschungszentrum and Charité-Universitätsmedizin Berlin, Berlin, Germany; 2 Department of Pathobiology, University of Pennsylvania School of Veterinary Medicine, Philadelphia, Pennsylvania, United States of America; 3 Research Center ImmunoSciences (RCIS), Charité-Universitätsmedizin Berlin, Berlin, Germany; 4 Department of Experimental Neurology, Charité-Universitätsmedizin Berlin, Berlin, Germany; 5 Robert Koch-Institut, Berlin, Germany; 6 Department of Microbiology and Immunology, University of Arkansas for Medical Sciences, Little Rock, Arkansas, United States of America; 7 Department of Inflammation Research, Amgen Inc., Thousand Oaks, California, United States of America; Johns Hopkins University - Bloomberg School of Public Health, United States of America

## Abstract

Infection with influenza virus can result in massive pulmonary infiltration and potentially fatal immunopathology. Understanding the endogenous mechanisms that control immunopathology could provide a key to novel adjunct therapies for this disease. Here we show that the cytokine IL-27 plays a crucial role in protection from exaggerated inflammation during influenza virus infection. Using *Il-27ra* ^−/−^ mice, IL-27 was found to limit immunopathology, neutrophil accumulation, and dampened T_H_1 or T_H_17 responses via IL-10–dependent and -independent pathways. Accordingly, the absence of IL-27 signals resulted in a more severe disease course and in diminished survival without impacting viral loads. Consistent with the delayed expression of endogenous *Il-27p28* during influenza, systemic treatment with recombinant IL-27 starting at the peak of virus load resulted in a major amelioration of lung pathology, strongly reduced leukocyte infiltration and improved survival without affecting viral clearance. In contrast, early application of IL-27 impaired virus clearance and worsened disease. These findings demonstrate the importance of IL-27 for the physiological control of immunopathology and the potential value of well-timed IL-27 application to treat life-threatening inflammation during lung infection.

## Author Summary

Annual epidemics of influenza result in 3 to 5 million cases of severe illness and approximately 300,000 deaths around the world. Although most patients infected with normal circulating influenza A viruses recover from the illness, complications arise during infections with highly pathogenic strains of the virus, resulting in increased mortality associated with severe immunopathology and acute respiratory distress. Previous studies suggested a major contribution of the vigorous immune response to lung damage. How the immune system constrains the negative impact of inflammation might therefore be of significant importance for future therapies. Our study in a mouse model of influenza shows that the cytokine IL-27 plays a crucial role in survival by protecting against lung damage. Its actions include regulation of innate (neutrophil influx) and adaptive (inflammatory cytokine production of T cells) arms of immunity during the acute respiratory infection. The data also suggest a therapeutic potential of IL-27, as mice treated with recombinant cytokine at later stages of infection exhibited decreased immunopathology and showed improved survival. The findings uncover an important role of IL-27 in limiting the collateral damages of anti-viral immunity and provide initial evidence that these mechanisms might be exploited for the management of severe immunopathology after infection.

## Introduction

Infection with highly pathogenic strains of influenza viruses, such as the pandemic 1918 Spanish flu, which resulted in 30–50 million deaths, is still a major threat to health [1], [2]. Pathological findings suggest that the vigorous mobilization of innate and adaptive arms of host immunity upon infection leads to uncontrolled inflammation and potentially fatal lung injury [3]. Rapid leukocyte infiltration of the lung and a subsequent cytokine storm involving the excessive production of inflammatory cytokines and chemokines have been strongly implicated in mediating lung immunopathology [3]–[5]. A better understanding of the factors that regulate the balance between viral clearance, tissue damage and resolution of inflammation is therefore necessary [6].

Interleukin 27 (IL-27) might be one important player in this context. The heterodimeric IL-27 belongs to the IL-12 superfamily and is composed of the Epstein-Barr virus inducible gene-3 (EBI3) and the IL-27p28 subunit [7]. The IL-27 receptor complex consists of IL-27Rα (WSX-1, TCCR) and the gp130 subunit, and is expressed by a wide range of cell types including T cells, monocytes, and neutrophils [8]. Initially, IL-27 was thought to promote T_H_1 responses because of its ability to induce T-bet expression, thereby triggering the upregulation of IL-12β2 receptor and IFN-γ under some conditions [9]–[11]. However, a series of subsequent studies using *in vivo* models of infection or autoimmune diseases provided evidence that its dominant function is rather to limit immune-mediated pathology [12].

Mice deficient in IL-27 receptor displayed increased immunopathology associated with overwhelming T_H_1 responses following infection with a number of parasites and intracellular bacteria [13]–[18]. Moreover, the lack of IL-27 receptor signaling resulted in augmented IL-17 production by CD4^+^ T cells in several animal models, including experimental autoimmune encephalomyelitis (EAE) [15], [19], [20]. Most studies have characterized the ability of IL-27 to suppress CD4^+^ T cell responses, but accumulating evidence suggests that the regulatory function of IL-27 also extends to cells of the innate immune system [8]. Consistent with its regulatory function, IL-27-mediated activation of STAT1, STAT3, STAT4 or BLIMP-1 promotes IL-10 and suppresses IL-17 production by CD4^+^ T cells [15], [21]–[23]. Additionally, IL-27 induced the expression of SOCS3 in CD4^+^ T cells, resulting in reduced IL-2 secretion in these cells [24].

The role of IL-27 in influenza has not been comprehensively studied. Liu *et al.* reported that influenza virus infection of epithelial cells or leukocytes induced IL-27, which correlates with increased serum levels of IL-27 in influenza patients [25]. Additionally, they showed a STAT1-dependent antiviral action of IL-27 *in vitro* [25]. Mayer *et al.* reported that IL-27 induces IFN-γ in transgenic CD8^+^ T cells [26]. In contrast, Sun *et al.* found no effect on T cell derived IFN-γ but a reduced IL-10 production by CD8^+^ T cells and increased leukocyte infiltration in infected *Ebi-3^−/−^* or conditional *Prdm1^−/−^* mice [23], [27].

We therefore investigated the impact of IL-27 and its receptor IL-27Rα on immunopathology using the highly mouse pathogenic [28] strain A/PR/8 (H1N1) and subsequently explored the therapeutic potential of recombinant IL-27 (rIL-27) to treat inflammatory lung disease in influenza. We found that, *Il-27ra^−/−^* mice exhibited increased mortality after influenza virus infection due to exaggerated immunopathology, in conjunction with augmented numbers of IFN-γ or IL-17-producing CD4^+^ and CD8^+^ T cells, and a strongly increased neutrophil infiltration. These effects were only partially attributed to diminished IL-27-induced IL-10. Thus, IL-27 plays an important role in limiting destructive inflammation, notably in the resolving phase of infection. Well-timed treatment with rIL-27 improved lung injury and accelerated recovery without affecting viral clearance. Our findings suggest that therapeutic application of rIL-27 predominantly suppresses innate cell recruitment but hardly affects the T cell response in the local tissue. These data demonstrate that IL-27 has a unique role in controlling immunopathology without impacting on host defense, and might therefore represent a promising candidate for immunomodulatory therapy of viral pneumonia.

## Results

### Impaired IL-27Rα signaling leads to increased mortality following influenza virus challenge

**Figure 1:**
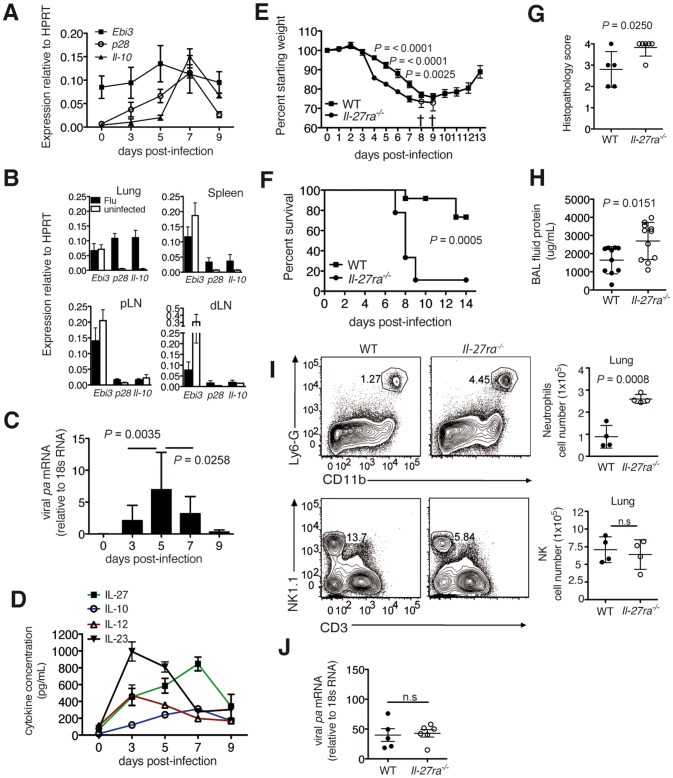
Absence of IL-27Rα leads to increased mortality and immunopathology during influenza. C57BL/6 mice were infected with a sublethal dose influenza virus. *Ebi3*, *Il-27p28* and *Il-10* mRNA in the (**A**) lung at indicated d.p.i or (**B**) spleen, peripheral (pLN) and lung-draining (dLN) lymph nodes of infected or uninfected C57BL/6 mice at 7 d.p.i. (**C**) Influenza virus polymerase (*pa*) mRNA expression or (**D**) cytokine concentration in the lung homogenate of infected C57BL/6 mice were analyzed at indicated d.p.i. (**E**) Weight loss or (**F**) survival of infected *Il-27ra^−/−^* (*n* = 9) or wild-type (WT) C57BL/6 (*n* = 12) mice after challenge with 3000 EID influenza virus. Open circles in **E** represent remaining live *Il-27ra^−/−^* mice (*n* = 2). (**G**) Pathological scores of H&E-stained lungs of *Il-27ra^−/−^* mice after 7 d.p.i. with 2500 EID influenza virus (**H**). Protein content in the BAL fluid of *Il-27ra^−/−^* mice at 9 d.p.i. was quantified by BCA. (**I**) Representative FACS plots and numbers of lung-infiltrating neutrophils or NK cells of *Il-27ra^−/−^* at 8 d.p.i. (**J**) Viral *pa* mRNA in lungs of *Il-27ra^−/−^* mice at 7 d.p.i was analyzed by qRT-PCR. Lung homogenates are a 20-fold dilution of homogenized whole lung tissue. All data sets were pooled from at least two independent experiments. Values represent means ± s.d. except for E, s.e.m. *P* values for **F** were determined by log-rank survival test. *P* values for **C**, **E**, **G**, **H**, **I** and **J** were determined by unpaired two-tailed Student's *t* test. ns, not significant.

To determine the role of IL-27 in shaping the immune response against influenza virus, we first examined the kinetics of *Il-27p28* and *Ebi3* mRNA expression in the lungs of sublethally infected C57BL/6 mice (**Fig. 1A**). While *Ebi3* was constitutively expressed and not significantly upregulated in the lungs and other organs (**Fig. 1B**), *Il-27p28* expression displayed a pronounced peak on day 7 post-infection (d.p.i.), two days after the peak of the viral load (**Fig. 1C**). Coinciding with the peak of *Il-27p28* expression was the maximal expression of *Il-10* mRNA, which is consistent with the assumption that IL-27 is an important inducer of IL-10 [15]. These mRNA data were confirmed at the protein level where IL-27 and IL-10 peaked at 7 d.p.i (**Fig. 1D**). In contrast, the inflammatory cytokines IL-12 and IL-23 were maximal already at 3 d.p.i (**Fig. 1D**). Thus, the expression kinetic of IL-27 in the infected lungs follows, with some delay, the kinetic of the virus load, being highest when virus is already declining and coming down when immunopathology has resolved. This is compatible with its role for dampening uncontrolled inflammation in a late phase while initially allowing for a rapid start of immune defense.

To assess the impact of IL-27 on survival during influenza, we challenged wild-type (WT) C57BL/6 or IL-27 receptor-deficient (*Il-27ra^−/−^*) mice with 3000 egg infectious dose (EID) influenza virus. *Il-27ra^−/−^* mice displayed accelerated weight loss and increased mortality following infection (**Fig. 1E, F**). Accordingly, *Il-27ra^−/−^* mice displayed a more severe lung pathology compared to control mice at 7 d.p.i (using a slightly lower virus dose, 2500 EID, to allow survival of all mice) (**Fig. 1G**). Furthermore, *Il-27ra^−/−^* mice had increased capillary leakage in the respiratory tract, leading to increased protein content in the bronchoalveolar lavage (BAL) fluid of these mice (**Fig. 1H**). A higher neutrophil, but not NK cell infiltration was observed in the lungs of *Il-27ra^−/−^* mice at 8 d.p.i (**Fig. 1I**). Remarkably, the increase in immunopathology and mortality in *Il-27ra^−/−^* mice was not due to a compromised viral elimination, as virus load was not significantly different between *Il-27ra^−/−^* and control mice (**Fig. 1J**). These findings demonstrate that IL-27 plays a critical role in limiting immunopathology during the later stages of infection.

### Uncontrolled IFN-γ and IL-17 T cell production in influenza virus infected *Il-27ra^−/−^* mice

**Figure 2:**
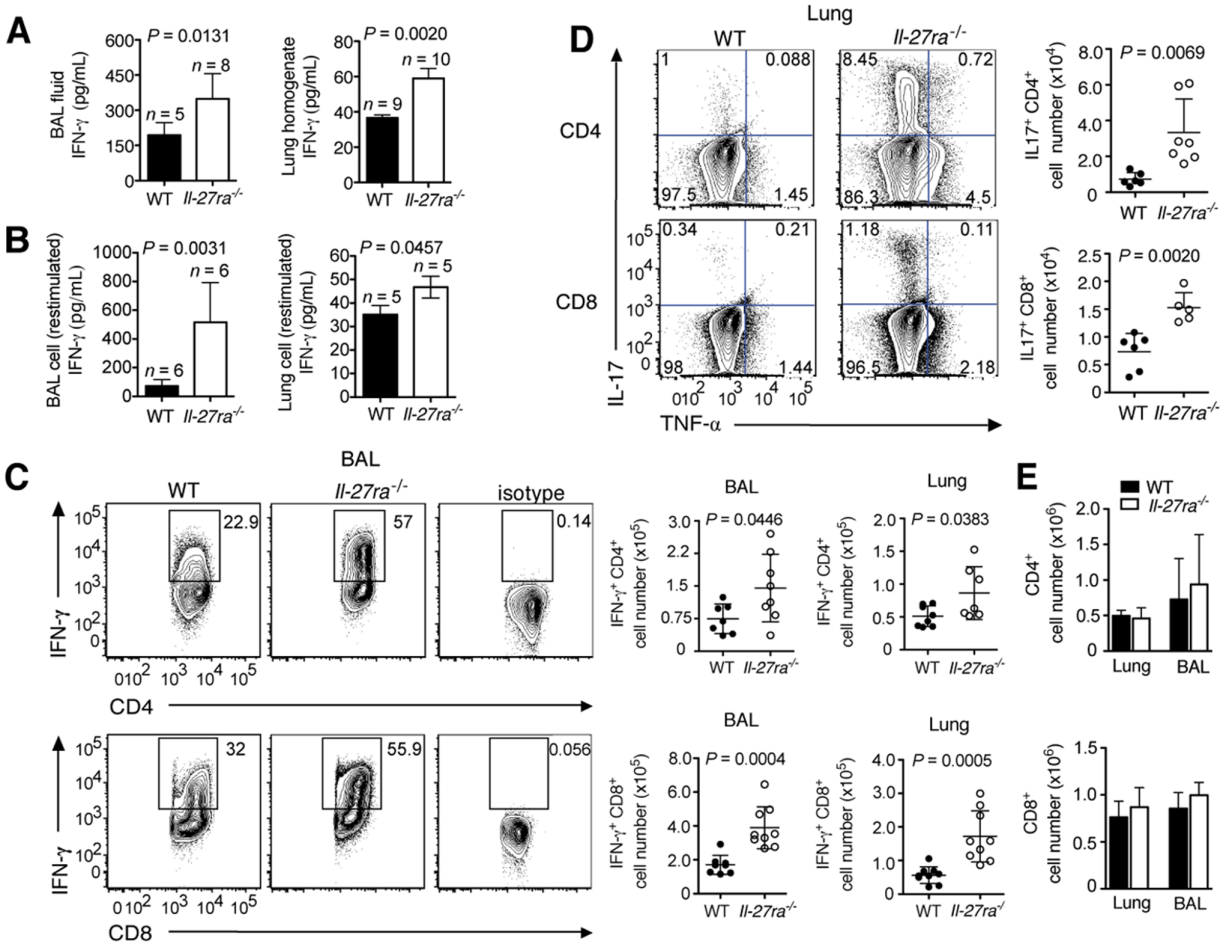
Absence of IL-27Rα leads to increased numbers of IFN-γ or IL-17-producing T cells in the respiratory tract. *Il-27ra^−/−^* or C57BL/6 mice were infected with 2500 EID influenza virus. At 9 d.p.i. IFN-γ concentrations in the (**A**) BAL fluid and lung homogenate of *Il-27ra^−/−^* mice or (**B**) supernatants of enriched *Il-27ra^−/−^* lymphocytes after PMA/ionomycin restimulation was quantified by ELISA. Numbers of (**C**) IFN-γ^+^ T cells in the BAL or lungs and (**D**) IL-17^+^ T cells from the lung were analyzed by FACS after PMA/ionomycin restimulation. (**E**) Total numbers of CD4^+^ or CD8^+^ T cells in the lungs and BAL of infected *Il-27ra^−/−^* and WT mice. Lung homogenates are a 20-fold dilution of homogenized whole lung tissue. All data sets were pooled from two independent experiments with similar results. *P* values were determined by unpaired two-tailed Student's *t* test. Values are means ± s.d.

Consistent with the ability of IL-27 to suppress T_H_1 and T_H_17 responses [15], [29], influenza virus infected *Il-27ra^−/−^* mice exhibited significantly increased IFN-γ levels in BAL fluid, lung homogenate (**Fig. 2A**), and supernatants of enriched lymphocytes from *Il-27ra^−/−^* mice after polyclonal stimulation using PMA/ionomycin (**Fig. 2B**). Accordingly, we detected increased numbers of CD4^+^ and CD8^+^ T cells able to produce IFN-γ upon re-stimulation in the BAL and lungs of the *Il-27ra^−/−^* mice (**Fig. 2C**). In contrast, IFNα levels in the BAL of infected *Il-27ra^−/−^* mice were not different from WT animals (**Fig. S1A**). The results suggest that IL-27 dampens IFN-γ-production by T cells during influenza.

Similar to the increased numbers of IFN-γ^+^ T cells, we observed augmented numbers of IL-17^+^CD4^+^, IL-17^+^CD8^+^ (**Fig. 2D**) and TNF-α^+^CD4^+^ T cells (**Fig. S1B**) in the lungs of infected *Il-27ra^−/−^* mice. A slight but not significant increase was also found for IL-4^+^CD4^+^ T cells (**Fig. S1C**). Total numbers of CD4^+^ and CD8^+^ T cells in the lungs at 9 d.p.i were not different between *Il-27ra^−/−^* and WT mice (**Fig. 2E**). Taken together, these data demonstrate that endogenous IL-27 limits the magnitude of effector cytokine production by T cells during influenza.

### Production of IL-10 by CD4^+^ T cells is induced by IL-27

**Figure 3:**
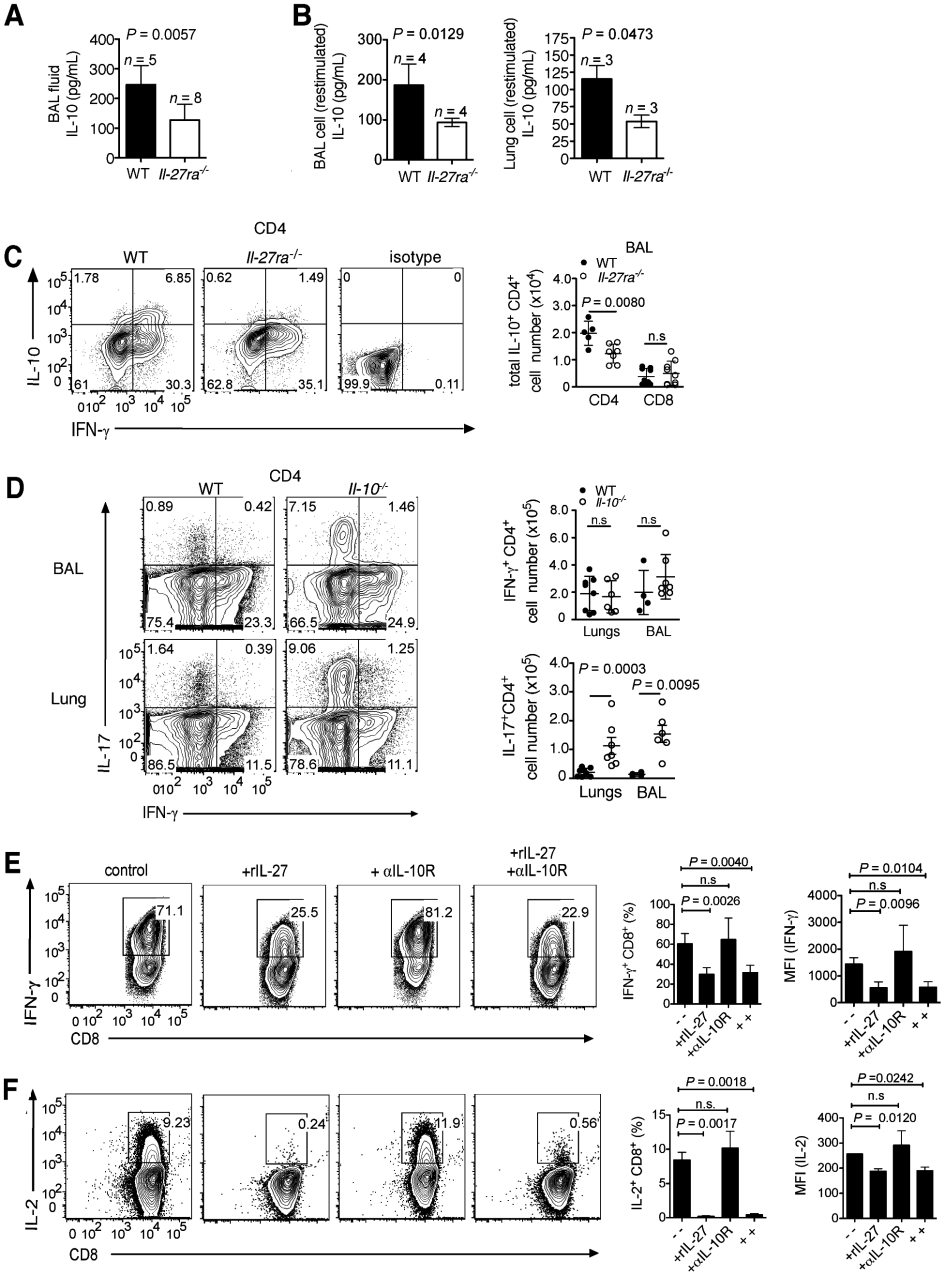
IL-27 directly modulates IFN-γ production by T cells and indirectly regulates IL-17 response via IL-10. *Il-27ra^−/−^* and C57BL/6 mice were infected with 2500 EID influenza virus. At 9 d.p.i, IL-10 levels in the (**A**) BAL fluid or (**B**) supernatants of enriched lymphocytes after PMA/ionomycin restimulation were determined by ELISA. (**C**) Total numbers of IL-10^+^ T cells in the BAL were analyzed by FACS after PMA/ionomycin restimulation. Representative FACS plots shown are gated CD4^+^ T cells from BAL. (**D**) *Il-10^−/−^* or C57BL/6 (WT) mice were infected with a sublethal dose influenza virus. At 7 d.p.i., influenza virus peptide-specific IFN-γ or IL-17-producing CD4^+^ T cells in the respiratory tract were assessed by FACS. (**E and F**) Naive CD8^+^ T cells were activated with plate-bound anti-CD3 and anti-CD28 in Tc1 polarizing conditions in the presence or absence of rIL-27 and/or anti-IL-10 receptor blocking antibody (αIL-10R). After 3 days (d), cells were transferred to plates, then media was replenished with rIL-2, rIL-27 and/or αIL-10R antibody for additional 2 d, for a total of 5 d in culture. IFN-γ or IL-2 production by Tc1 cells were analyzed by FACS. **A** to **D** were pooled from two independent experiments with similar results. **E** and **F** represent data from three independent experiments with similar results. *P* values were determined by unpaired two-tailed Student's *t* test. Values are means ± s.d. ns, not significant.

Consistent with the role of IL-27 in inducing IL-10 production by CD4^+^ T cells in other models [15], [30]–[32], infected *Il-27ra^−/−^* mice had decreased levels of IL-10 in the BAL fluid (**Fig. 3A**) and in the supernatant of PMA/ionomycin-restimulated lymphocytes (**Fig. 3B**). This decrease correlated with the impaired ability of *Il-27ra^−/−^* CD4^+^ T cells from infected mice to produce IL-10 after *in vitro* restimulation with PMA/ionomycin (**Fig. 3C**). Moreover, *Il-27ra^−/−^* mice had reduced numbers of IL-10^+^IFN-γ^+^ double-positive cells, while total IFN-γ^+^CD4^+^ T cells were increased, resulting in a significantly reduced IL-10:IFN-γ ratio in CD4^+^ T cells of *Il-27ra^−/−^* mice compared to WT animals (**Fig. S2**). Although reduced, IL-10-producing CD4^+^ T cells were not completely lacking in *Il-27ra^−/−^* mice, suggesting that other factors besides IL-27 contribute to the induction of IL-10 [33].

Numerous studies have established that IL-27 is signaling via STAT factors such as STAT1, STAT3 and STAT4. Among these, STAT4 has been shown to be involved in the induction of IL-10 production by CD4^+^ T cells [22], [34]. STAT4 is, however, also the main intermediate of IL-12 signaling. Sublethal influenza virus infection of *Stat4^−/−^* mice resulted in significantly fewer lung-infiltrating IL-10^+^IFN-γ^+^CD4^+^ T cells; yet this was not the case in *IL-12p40^−/−^* mice. Thus, IL-27 but not IL-12 is responsible for STAT4 mediated induction of IL-10 (**Fig. S3A, B**). Viral loads in the lungs of infected *Stat4^−/−^* mice at 9 d.p.i were not significantly different to WT animals (**Fig. S3C**).

Thus, IL-10 becomes induced in IFN-γ^+^CD4^+^ T cells during influenza by IL-27, in part mediated via STAT4.

### IL-27 regulates IL-17 via IL-10 and directly modulates IFN-γ production in T cells

We next determined whether IL-10 mediates the anti-inflammatory effects of IL-27. To this end, we infected *Il-10^−/−^* mice with influenza virus and analyzed influenza peptide-specific IL-17 or IFN-γ-producing T cells. Indeed, *Il-10^−/−^* mice had elevated numbers of IL-17^+^CD4^+^ (**Fig. 3D**) and a slightly increased numbers of IL-17^+^CD8^+^ T cells in the lungs (**Fig. S4**), similar to that of infected *Il-27ra^−/−^* mice. These results indicate IL-17 suppression is largely mediated via IL-10.

In contrast, the increased numbers of IFN-γ-producing CD4^+^ or CD8^+^ T cells in the respiratory tract of infected *Il-27ra^−/−^* mice were not observed in *Il-10^−/−^* mice (**Fig. 3D** and **Fig. S4**). These findings were verified by blocking IL-10 signaling *in vivo* by administration of an anti-IL-10 receptor antibody (αIL-10R; **Fig. S5A, B**). Viral titers in the lungs of *Il-10^−/−^* mice were not different to that of WT mice (**Fig. S5C**).

The ability of IL-27 to directly modulate IFN-γ production in CD8^+^ T cells was confirmed *in vitro*, where addition of rIL-27 to the cultures strongly suppressed IFN-γ and IL-2 production by activated IFN-γ^+^CD8^+^ T cells (Tc1 cells), even when IL-10R signaling was blocked (**Fig. 3E, F** and **Fig. S6**). An IL-27-dependent suppression of IFN-γ and IL-2 in CD4^+^ T cells had already been described previously [21].

These results demonstrate that IL-10-dependent effects only partially account for IL-27 mediated suppression. Notably the IL-10-independent effects on IFNγ-production, but also on distinct recruitment events (see below) might explain why deficiency in IL-27 signaling has a strong impact on the disease course in influenza while IL-10 deficiency has not (**Fig. S7**; for the latter see also [35]).

### Treatment with recombinant IL-27 alleviates immunopathology when administered in a late phase of the infection

**Figure 4:**
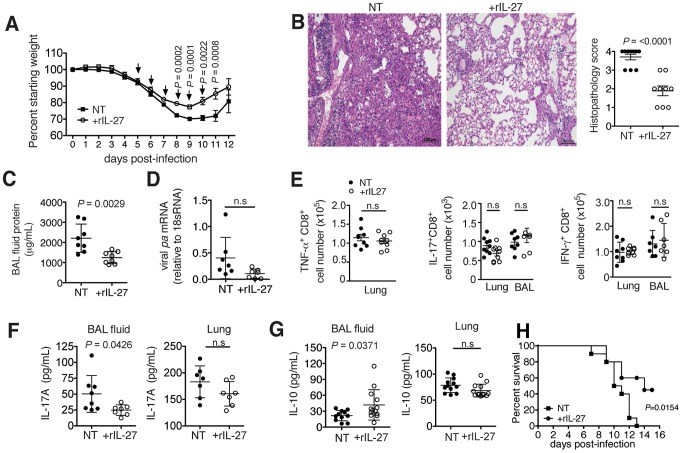
Late-phase treatment with rIL-27 alleviates lung immunopathology. C57BL/6 mice were challenged with a sublethal (A to G) or lethal (H) dose of influenza virus and treated daily with rIL-27 from 5–10 (A, H) or 5–9 (B to G) d.p.i. Non-treated control mice (NT) were injected with PBS. (**A**) Weight loss of rIL-27-treated or NT mice. Arrows (↓) indicate points of treatment. At 9 d.p.i., (**B**) histological comparison of H&E-stained lungs was performed, (**C**) protein content in the BAL fluid was measured by BCA, (**D**) viral *pa* mRNA expression in the lungs was measured by qRT-PCR and (**E**) influenza virus peptide-specific cytokine production by CD8^+^ T cells was determined by FACS. Levels of (**F**) IL-17 and (**G**) IL-10 in the BAL fluid or lung homogenates of late-treated or NT mice at 9 d.p.i. were measured by ELISA. Lung homogenates are a 20-fold dilution of homogenized whole lung tissue. (H) Survival of mice treated with rIL27 or PBS from day 5–10 after challenge with a lethal dose of influenza virus. Values for weight loss curves are data pooled from at least two independent experiments, representing the means ± s.d. of the following numbers of mice per group: day 1–9, *n* = 14; day 10–11, *n* = 7; day 12, *n* = 6. Data from **B** to **H** are pooled from at least two independent experiments with similar results. *P* values were determined by unpaired two-tailed Student's *t* test. Values are means ± s.d. except for A, s.e.m.; ns, not significant. *P* values for **H** were determined by log-rank survival test (n = 10).

Having demonstrated the pronounced role of IL-27 in regulating immunopathology, we wondered whether this property could be exploited for therapeutic purposes. In accordance with the delayed endogenous production of IL-27, we administered exogenous rIL-27 from 5–10 d.p.i. Indeed, this treatment regimen resulted in decreased weight loss and accelerated recovery (**Fig. 4A**), a striking improvement in lung immunopathology (**Fig. 4B**), and in reduced capillary leakage as indicated by a lower BAL fluid protein content (**Fig. 4C**). Notably, rIL-27 therapy did not impair viral clearance (**Fig. 4D**). In line with this, the numbers of CD8^+^ (**Fig. 4E**) or CD4^+^ (**Fig. S8**) T cells from the infected respiratory tract producing either TNF, IL-17 or IFN-γ upon antigen-specific stimulation were not changed. Only a slight decrease of secreted IL-17 (**Fig. 4F**) and increase of IL-10 levels (**Fig. 4G**) was found in the BAL fluid. The protective effect of treatment with rIL-27 was also found when mice were infected with a lethal dose of influenza virus (**Fig. 4H**). We did not observe significant effects of IL-27 treatment on the activity of virus-specific CTLs as measured by the CD107 mobilization assay or the fraction of IFN-γ-producing CD8+ cells (**Fig. S8**).

**Figure 5:**
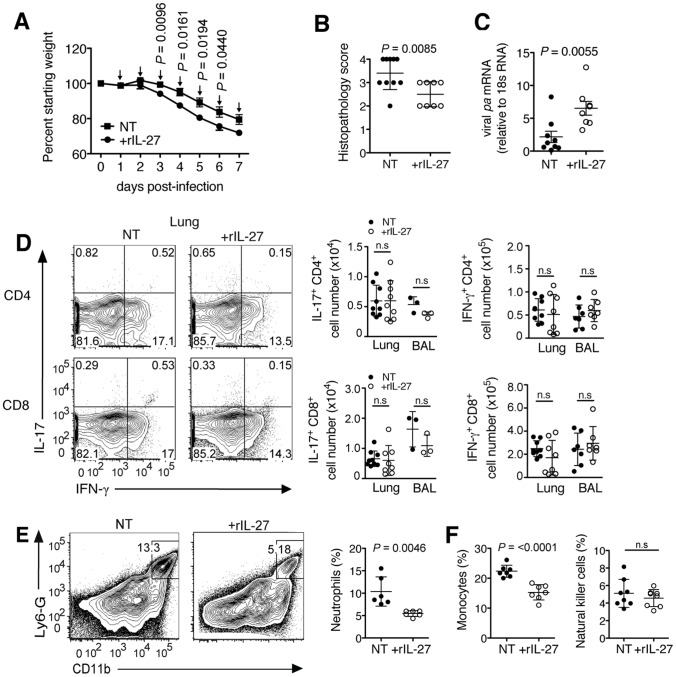
rIL-27 treatment at an early stage of infection aggravates disease severity and impairs viral clearance. C57BL/6 mice were challenged with a sublethal dose influenza virus then treated daily with rIL-27 from 1–7 d.p.i. Non-treated control mice (NT) were injected with PBS. (**A**) Weight loss of rIL-27 treated (*n* = 5) or NT (*n* = 5) mice. Arrows (↓) indicate time of treatment. At 7 d.p.i., (**B**) histological scores of H&E-stained lungs from treated or NT groups and (**C**) viral *pa* mRNA expression were determined. (**D**) Numbers of influenza virus peptide-specific IL-17 or IFN-γ-producing T cells, (**E**) influx of neutrophils, (**F**) monocytes and NK cells in the lungs at 9 d.p.i. were determined by FACS. Gated cells in FACS plots in **e** indicate the percentage of neutrophils from total lung cells. Data from **A** are representative of two independent treatment experiments. Data from **B** to **F** are pooled from at least two independent experiments. *P* values were determined by unpaired two-tailed Student's *t* test. Values are means ± s.d. except for A, s.e.m.; ns, not significant.

To test whether the delayed kinetics of endogenous IL-27 is relevant for an unhindered initial response to infection, we applied exogenous rIL-27 from 1–7 d.p.i. (early phase). Mice treated under this regimen exhibited stronger weight loss (**Fig. 5A**) and reached the limits for euthanasia at 7 d.p.i. To assess the impact of treatment on immunopathology and other parameters, all animals were sacrificed at this time point. Although a diminished lung histopathology was observed (**Fig. 5B**), a significantly higher viral load was found (**Fig. 5C**). Impaired viral clearance was not due to a suppressed T cell cytokine response as treated mice had unchanged numbers of influenza peptide specific IL-17^+^ or IFN-γ^+^ T cells in the respiratory tract (**Fig. 5D**). However, mice treated in the early phase with rIL-27 had significantly reduced frequencies of neutrophils (**Fig. 5E**) and monocytes, but not NK cells (**Fig. 5F**). In our model, NK cells played a minimal role in viral clearance, as NK1.1 cell-depletion did not influence viral loads (**Fig. S9**).

These findings suggest that systemic rIL-27 treatment during the early stages of influenza has little impact on the local antigen-specific T cell response, but suppresses neutrophil and monocyte influx that are crucial for the control of infection at this stage [28], [36]. In contrast, treatment at a later time-point, starting at the peak of viral load, did not impair viral clearance but immunopathology and disease course were markedly improved.

### IL-27 treatment works by suppressing leukocyte recruitment into the infected lungs

**Figure 6:**
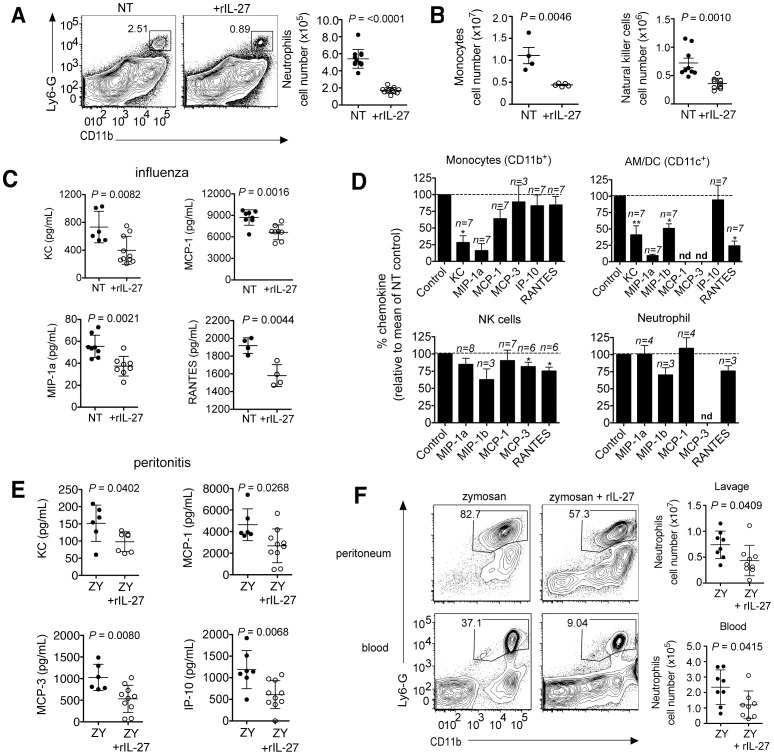
Late IL-27 treatment suppresses innate cell migration and chemokine production by CD11b^+^ and CD11c^+^ cells. C57BL/6 mice were challenged with a sublethal dose of influenza virus then treated daily with rIL-27 from 5–9 d.p.i. At 9 d.p.i., (**A**) numbers of neutrophils, (**B**) monocytes or NK cells in the lungs were analyzed by FACS. Chemokine concentration in the (**C**) BAL fluid of late IL-27-treated influenza virus infected mice. (**D**) Chemokine levels in cultures of CD11b^+^, CD11c^+^, NK (NK1.1^+^CD3^−^) cells or neutrophils (Ly6G^+^CD11b^+^) isolated from influenza virus-infected lungs after a 24 h treatment with rIL-27. (**E**) C57BL/6 mice were co-injected i.p. with zymosan (ZY) and rIL-27. Injection of zymosan only was used as control. After 24 hours, chemokine levels in the peritoneal fluid were analyzed and (**F**) the numbers of neutrophils in the peritoneum and blood were determined by FACS and. All data sets are pooled from at least two independent experiments with similar results. *P* values were determined by unpaired two-tailed Student's *t* test. Values in **A**, **B**, **C**, **E** and **F** are means ± s.d.; **D** indicate means ± s.e.m. *P<0.05; **P<0.01; nd, not detected.

Therapeutic application of rIL-27 from 5–9 d.p.i. had surprisingly little impact on the T cell compartment, but strongly suppressed the accumulation of neutrophils (**Fig. 6A**), monocytes and partially NK cells in the lung (**Fig. 6B**). We therefore conclude that IL-27 can regulate innate cell trafficking independently of any effect on T cell responses. Reduction of neutrophils, but not of NK cells was mediated via IL-10, as is the reduction of some chemokines in the BAL (Fig. **S12**)

Leukocyte accumulation in sites of inflammation is regulated by chemokines and adhesion molecules governing both the entry and exit from tissue. Indeed, rIL-27-treatment during influenza reduced the levels of multiple chemokines in the BAL fluid (**Fig. 6C**). To identify the cellular targets of IL-27-dependent chemokine suppression, different leukocyte subsets from the lungs of influenza virus infected mice were isolated and cultured overnight in the absence or presence of rIL-27. IL-27 suppressed chemokine production by CD11b^+^ or CD11c^+^ cells but had little impact on NK cells or neutrophils (**Fig. 6D**). Especially the chemokines KC (CXCL1), MIP-1β (CCL4) and RANTES (CCL5), which are prototypic attractors of neutrophils, monocytic, and lymphocytic cells, were suppressed. A similar suppression by IL-27 was found for IL-1β or IL-6-induced chemokine production by endothelial cells isolated from the lungs (**Fig. S10**).

To confirm the ability of IL-27 to suppress leukocyte recruitment in the absence of a significant contribution from T cells, we determined the impact of IL-27 treatment in the T cell-independent zymosan-induced peritonitis model. Similar to influenza, rIL-27 significantly reduced the levels of chemokines in the peritoneal lavage as well as the numbers of neutrophils in peritoneum and blood (**Fig. 6E, F**). As only minimal levels of IL-17 are present in the peritoneal lavage (**Fig. S11**), this effect does not rely on IL-17 suppression by IL-27. These data unravel a novel mode of action of IL-27 that is based on suppression of innate cell recruitment into sites of inflammation.

## Discussion

The tight regulation of both the induction and subsequent down-regulation of inflammatory responses during influenza is imperative in minimizing severe immunopathology. Infection with highly pathogenic strains of influenza viruses results in increased leukocytic pulmonary infiltrates and leads to the exaggerated production of inflammatory cytokines (“cytokine storm”) that causes massive inflammation with increased mortality [4], [5], [37]. Therefore, understanding the regulatory pathways during infection not only sheds light on the mechanisms controlling the delicate balance of efficient viral clearance and disastrous immunopathology, but also reveals potential therapeutic approaches to target resolution of inflammation [6]. Few studies have evaluated the therapeutic potential of anti-inflammatory agents in influenza; while broad-acting immunosuppressants such as corticosteroids were found to worsen the disease, a combination of antiviral therapy and anti-inflammatory non-steroidals inhibiting cyclooxygenases (COX) improved survival in mice [38]. Similarly, targeting inhibitory pathways such as macrophage CD200, PAR_2_ and endothelial S1P_1_ receptors have been found to reduce immunopathology in influenza infection models [39]–[41]

Our findings suggest that IL-27 is a potential candidate for the treatment of immunopathology, as endogenous IL-27 was found to play a major role in dampening of exaggerated inflammation in influenza while having little impact on virus elimination. The absence of IL-27Rα signaling during acute virus infection worsened immunopathology and disease course; this ultimately resulted in increased mortality, despite controlled viral loads. Additionally, increased neutrophil accumulation and augmented IFN-γ or IL-17 production by T cells were observed in the infected *Il-27ra^−/−^* mice while local IFNα levels appeared not to be affected. These data are in agreement with a number of *in vivo* models of bacterial or parasitic infection that underline a crucial role of IL-27 in dampening inflammation [14], [16]–[18], [42].

That IL-27 acts *in vivo* predominantly as an anti-inflammatory cytokine was not foreseen in the beginning, as several studies demonstrated activating effects of IL-27, e.g. on the production of IFN-γ *in vitro* [9], [10], [43]. In an influenza model, Mayer *et al.* reported that WT CD8^+^ T cells displayed higher IFN-γ production than IL-27Rα-deficient cells [26]. In this chimera model, non-hematopoietic and half of the hematopoietic cells responded to IL-27 so that only T cell- intrinsic effects of deficiency were effective. In contrast, under the conditions of global absence of IL-27Rα as used here, we observed increased IFN-γ levels and two-fold higher numbers of IFN-γ^+^ T cells in the infected respiratory tract of *Il-27ra^−/−^* mice, in line with the findings of the above-mentioned parasite infection models. We assume, that the global effect of IL-27 *in vivo* involves a complex network of cell types including myeloid cells or even non-hematopoietic cells. In addition, timing and conditions might be crucial for the quality of IL-27 effects, as we also found a direct, IL-10-independent suppression of IFN-γ and IL-2 in activated Tc1 cells by IL-27 *in vitro*. Thus, the environmental context plays a significant role for the action of IL-27 *in vivo*, and its impact on the innate response might dominate over effects restricted to the T cell compartment. Indeed, the strong increase in the number of lung-infiltrating neutrophils in the absence of IL-27 signaling was one of the most impressive findings and appears to be crucial for the worsened immunopathology.

Major effects of compromised IL-27 signaling were also found on the number of IL-17 producing T cells. Both IFN-γ and IL-17 have been reported to play a significant role for lung injury during influenza [4], [44], [45]. IL-17 has been described as a major factor boosting expansion, recruitment and activation of neutrophils by inducing hematopoietic growth factors, chemokines and other activating signals [46]–[48]. IL-27-dependent regulation of T_H_17 responses was reported to occur through a number of mechanisms [15], [20], [49]–[51]. Here we provide evidence that suppression of IL-17 is largely dependent on IL-10 acting as an intermediate, since infected *Il-10^−/−^* mice displayed augmented numbers of IL-17^+^ T cells, similar to that observed in *Il-27ra^−/−^* mice. This is in agreement with a previous study in which blocked IL-10R signaling during high dose influenza virus infection resulted in elevated numbers of IL-17^+^CD4^+^ T cells [35]. In contrast, IFN-γ-producing T cells were not affected by absence of IL-10.

Based on these data demonstrating the important role of IL-27 in controlling inflammation, we reasoned that application of rIL-27 might be of value in situations in which exaggerated immunopathology, rather than virus elimination, becomes a critical issue for host survival as it is often the case in severe influenza. Indeed, systemic application of daily doses of rIL-27 at 5–9 d.p.i accelerated recovery and alleviated immunopathology by suppressing the influx of neutrophils, monocytes and, to a lesser degree, NK cells into the infected lungs of mice. Reduced infiltration appears to be the major cause of the improved overall status of treated mice, as large numbers of these cells can contribute to lethal lung damage by producing inflammatory cytokines, chemokines and reactive oxygen species, which results in the amplification of inflammatory signals [4], [5], [37]. Again, the reduction in infiltrating neutrophils upon IL-27 therapy was largely dependent on IL-10 (**Fig. S12**). In contrast, the reduced infiltration of NK cells was not dependent on IL-10, underlining that not all effects of IL-27 are mediated by induced IL-10 and that IL-27 has a broader suppressive effect than its downstream-mediator IL-10. This latter conclusion is supported by the finding that infected *Il-27ra^−/−^*, but not *Il-10^−/−^* mice exhibited a more severe disease course compared to WT animals.

Surprisingly, the number and cytokine profile of influenza virus-specific T cells in the lung was not significantly affected by treatment with rIL-27. Moreover, virus elimination was not impaired, if not even improved, upon treatment in the late phase. Whether this is due to the reported induction of antiviral activity by IL-27 that activates an interferon-induced antiviral protein kinase-R (PKR) via STAT1 in human lung epithelial cells [25], or whether destructive inflammation counteracts an efficient antiviral defense, remains to be shown.

Although some reduction in the level of IL-17 and increase in IL-10 was found in the BAL fluid upon treatment, these findings suggest that rIL-27 applied systemically predominantly regulates innate cell accumulation in the lungs rather than limiting the activity of the adaptive arm of the immune system such as IFN-γ or IL-17 producing T cells within the inflamed tissue. An explanation could be that local levels of IL-27 calculated for the lung tissue of infected WT animals are two orders of magnitude higher than plasma levels after systemic application of rIL-27 and are therefore hardly increased upon treatment (**Fig. S13**). We therefore propose that systemically applied rIL-27 predominantly acts on cells exposed directly to blood or plasma exudate and/or on innate cells before or during their journey to the inflamed lung.

To test the hypothesis that IL-27 treatment is able to suppress the accumulation of neutrophils independent of T cells, we applied rIL-27 in an acute model of TLR-induced sterile inflammation, the zymosan-induced peritonitis model. In this model, T cells are virtually absent in the inflammatory site, and IL-17 is hardly detectable. Indeed, rIL-27 inhibited the accumulation of neutrophils also under these conditions.

As leukocyte trafficking is controlled by adhesion molecules and chemokines presented on endothelial cells, we tested whether IL-27 affects key molecules involved in the recruitment or retention of innate leukocytes in influenza. Consistent with a role for IL-27 in modulating the trafficking of neutrophils and monocytes, treatment with rIL-27 reduced the production of neutrophil and monocyte chemoattractants KC (CXCL1), MIP-1α (CCL3), and RANTES (CCL5) produced *in vitro* by pulmonary monocytes (CD11b^+^), alveolar macrophages/dendritic cells (CD11c^+^) or NK cells isolated from infected lungs and resulted in reduced chemokine levels in the BAL fluid of infected mice. Similar effects were found with lung endothelial cells. These data complement recent findings that IL-27 suppresses the response of macrophages to TNF-α and IL-1 [52].

While the altered levels of chemokines in the BAL might affect the retention of leukocytes in the alveolar space, the deposition of chemokines on the endothelial surface by macrophages lining the blood vessels would directly affect the adhesion and transmigration of circulating leukocytes. Indeed, a major fraction of monocytic cells in the lung is not situated in the parenchyma but sitting within the vessel wall (“marginal pool”), rendering these cells sensitive to the cytokines in the blood, including exogenously administered cytokines [53], [54]. In addition we found that the chemokine production of endothelial cells upon stimulation with IL-1β or IL-6 was suppressed by IL-27. Moreover, IL-27 has been reported to directly affect adhesion and activation of neutrophils [55].

The expression of the IL-27p28 subunit in the influenza virus-infected respiratory tract peaks at the later phase of infection when viral titers are at a decline, which is consistent with the suggested role of IL-27 in limiting the immune response. Interferons can elicit IL-27 production as the *Il-27p28* gene promoter contains an IFN-stimulated response element region (ISRE), which becomes activated through IRF-1 [56]–[58]. In contrast to the inflammatory cytokines IL-12 or IL-23, which are rapidly produced by myeloid cells, e.g. upon triggering TLR receptors, and accordingly found in early time points in the influenza infection, the expression of IL-27 is turned on in a delayed fashion by the inflammatory microenvironment and serves as a negative feedback mechanism, thereby dampening the immune response in the later phase when adaptive immunity is established and the risk of severe immunopathology comes to the fore.

In line with this concept, we observed protective effects when rIL-27 was administered in a later phase of infection, starting at the peak of viral load when also the endogenous IL-27 production is near its highest level. To test whether timing is crucial, we additionally applied rIL-27 in the early phase of infection, starting 1 day after infection. Indeed, under these conditions IL-27 treatment also reduced leukocyte infiltration and immunopathology, but simultaneously impaired virus elimination, resulting in a worsened disease course. This suggests that interference with leukocyte recruitment in the early phase of influenza aggravates the infection, and the low level of endogenously produced IL-27 in this early phase is appropriate to allow their unhindered rapid activity in virus defense.

Indeed, previous studies have demonstrated that neutrophils are essential for early host protection in influenza infection, as neutrophil depletion before infection led to increased viral titers and accelerated mortality [28], [36]. The same was found for alveolar macrophages [36]. While their mode of action is still unknown, these studies suggest that neutrophils and macrophages contribute to protection in the early phase. In the late phase, depletion of neutrophils or macrophages was not affecting the disease course, while even infection with a low pathogenic strain was fatal in RAG-2γc^−/−^ mice lacking NK, T and B cells [28]. On the other side, recruited leukocytes have a major role in immunopathology, e.g. by inducing apoptosis in epithelial cells [59]. These findings are compatible with the paradigm that the innate system contributes to early protection in viral infection, while at later time points, antigen-specific T cells take over and eliminate the virus.

In conclusion, our study shows that endogenous IL-27 has a crucial role in preventing a fatal disease course in influenza where it acts to limit and resolve the inflammatory process while allowing an unimpaired antiviral response (**Fig. S14**). Based on its physiological role as a master factor regulating IL-10-dependent as well as -independent anti-inflammatory mechanisms, we here demonstrate that well-timed therapeutic application of recombinant IL-27 can successfully counteract detrimental immunopathology while keeping the antiviral response intact. Combination of IL-27 treatment with anti-viral or anti-microbial treatment might further expand the applicability of this concept, especially when the role of IL-27 in secondary bacterial infection [60] is appropriately taken into account.

These data suggest that strategies to target natural multifunctional pathways involved in the resolution of inflammation might be a valuable alternative for the treatment of inflammation-caused immunopathology and complement current therapeutic approaches focused on the inhibition of isolated effector mechanisms.

## Materials and Methods

### Mice and infection

Wild-type (WT) C57BL/6, BALB/c, *Il-10^−/−^, Il-12p40^−/−^* and *Stat4^−/−^* mice were purchased from Taconic Farms, Charles River, or The Jackson Laboratory. *Il-27ra^−/−^* mice were backcrossed more than nine generations to C57BL/6 mice [11] and housed in the Department of Pathobiology at The University of Pennsylvania, Philadelphia, USA. Mice were infected with influenza A/PR/8/34 virus intranasally (i.n) while under dexdomitor (0.5 mg/mL, Pfizer) and anti-mepazole (5 mg/mL, Pfizer) anesthesia, or with light isofluorane. For sublethal infection with influenza virus, BALB/c and *Stat4^−/−^* mice were infected with 12.5 plaque forming units (PFU), while C57BL/6, *Il-10^−/−^* and *Il-27ra^−/−^* mice were infected with 25 PFU or 2500 egg infectious dose (EID). Survival assays with *Il-27ra^−/−^* mice was performed with 3000 EID. For experiments addressing the therapeutic potential of rIL-27, 45 PFU of the virus were used as sublethal and 55 PFU as lethal dose. Infected mice were weighted daily and assessed for clinical symptoms of infection. When reaching a weight loss of >30%, mice were euthanatized. Animal care and experiments were performed in accordance with the institutional guidelines of German Federal Law and local authorities of Berlin (LAGESO), or Animal Care and Use Committee of the University of Pennsylvania.

### Ethics statement

Animal procedures were performed in accordance with the German “Tierschutzgesetz in der Fassung vom 18. Mai 2006 (BGBI.IS.1207)” and the guideline 2010/63/EU from the European Union and the European Convention for the protection of vertebrate animals used for experimental and other scientific purposes. Animal protocols were approved by the ethics committee and the Berlin state authorities (LAGeSo Registration # G03310/08). Experiments performed at the University of Pennsylvania were carried out in accordance with the guidelines in the Guide for the Care and Use of Laboratory Animals of the National Institutes of Health. The animal protocol (# 802004) was approved by the Institutional Animal Care and Use Committee (IACUC) of the University of Pennsylvania, Philadelphia PA (Federal assurance # FWA00004028; Office of Laboratory Animal Welfare assurance # A3079-01).

### Influenza virus

Influenza A/PR/8/34 virus was grown in the allantoic cavaties of 11-day old embryonated chicken eggs or was purchased from Charles River.

### Bronchoalveolar lavage fluid and tissue preparation

Bronchoalveolar lavage (BAL) was obtained by flushing the airways 3× with 1 mL sterile PBS. Lungs (approximately 200 mg of tissues) were mashed through a 70 µm cell strainer and suspended in 10 mL of PBS/BSA. Lung lymphocytes were enriched using a percoll gradient 40∶70 (vol/vol). Erythrocytes were lysed with an erylysis buffer (Sigma). BAL fluid and lung homogenates were collected and stored at −80°C for ELISA and BCA.

### Leukocyte preparation for chemokine determination

CD11b^+^, CD11c^+^, NK1.1^+^CD3^−^ (NK cells) and Ly6G^+^CD11b^+^ (neutrophils) cells were isolated from infected lungs of C57BL/6 mice at 5 d.p.i. and sorted by FACS. Cells (1×10^5^/50 µL cRPMI) were incubated in the presence or absence of rIL-27 (50 ng/mL) for 24 hours (h) without further stimulation and chemokine concentrations in supernatants analyzed.

### Assessment of lung injury, ELISA and multiple chemokine detection

Total BAL fluid protein was measured using a bicinchoninic protein assay (BCA) kit (Pierce Chemical). Cytokine or chemokine concentration in BAL fluid, lung homogenates or cell cultures were measured by enzyme-linked immunosorbent assay (BD Biosciences or eBiosciences) or FlowCytomix multiple detection kit (eBioscience).

### Zymosan-induced peritonitis

C57BL/6 mice were injected with zymosan (1 mg in 1 mL PBS) intraperitoneally (i.p) with or without co-injection of rIL-27 (200 ng, R&D Systems). After 24 h, blood or 5 mL peritoneal lavage (PBS) was obtained.

### Lymphocyte culture

Naive CD8^+^ T cells (CD8^+^CD62L^+^) were isolated from pooled splenocyte and lymph nodes using magnetic bead separation (Miltenyi). For activation, purified CD8^+^ T cells (1×10^6^ cells per mL) were cultured with plate-bound anti-CD3 (3 µg/mL; clone 145-2C11) and anti-CD28 (3 µg/mL; clone 37.51). CD8^+^ T cells were supplemented with murine rIL-12 (5 ng/mL; eBioscience), rIFN-γ (20 ng/mL; eBioscience), and rIL-2 (10 ng/mL; R&D Systems) plus anti-mouse IL-4 antibody (5 µg/mL; clone 11B11) in complete RPMI media (RPMI 1640 (Gibco®) plus 10% FCS (vol/vol) and antibiotics) for 3 days (d). After 3 d in culture, Tc1 cells were transferred to a plate without anti-CD3 and anti-CD28 and supplemented with fresh medium plus rIL-2 and cultured for 2 d for a total of 5 d in culture. In cases where rIL-27 (rIL-27) was added or IL-10 receptor was blocked, the media were supplemented with rIL-27 (50 ng/mL; eBioscience) and/or anti-IL-10 receptor blocking antibody (αIL-10R) (40 µg/mL; 1B1-2). In some experiments, cells were labeled with CFSE (Sigma) and cultured under the same conditions as mentioned above.

### T cell re-stimulation and intracellular cytokine staining

Intracellular cytokine staining (ICS) was performed as previously described (Hamada et al., 2009). Briefly, enriched lymphocyte samples were restimulated either with phorbol 12-myristate 13-acetate (PMA; 100 ng/mL) plus ionomycin for 4 h, or a combination of immunodominant influenza virus peptides (Anaspec; **Table S1**) for 6 h in the presence of Brefeldin A (10 µg/mL, Sigma). For analysis of CD107 expression, anti-CD107a antibody (10 µg/mL) and anti-FcRγ (10 µg/mL) was added to the culture during 6 h peptide restimulation. Cells were incubated with anti-FcRγ receptor antibody prior to staining for surface markers. Surface-labeled cells were fixed for 20 minutes using 2% paraformaldehyde. Cytokines were stained using fluorochrome-labeled anti-mouse monoclonal antibodies (mAbs) in 0.1% saponin buffer for 20 minutes. Cells were washed and resuspended in PBS/BSA then analyzed on a FACS Canto II (BD Biosciences). Data were analyzed using Flowjo analysis software (TreeStar). For FACS antibodies used, see supplemental methods.

### Quantitative reverse-transcription PCR

RNA was extracted using RNeasy Mini Kit with oncolumn DNase digestion (Qiagen). cDNA was synthesized using SuperscriptII Reverse Transcriptase (Invitrogen) with random hexamers and oligo(dT) primers (Qiagen). Quantitative reverse-transcription PCR (qRT-PCR) was performed using Platinum SYBR Green qPCR SuperMix-UDG (Invitrogen) on a Stratagene MX3000 thermo cycler. For qRT-PCR primers used, see **Table S2**.

### *In vivo* treatments

Blockade of IL-10 signaling *in vivo* was achieved by administration of an anti-IL-10 receptor-specific mAb (αIL-10R; clone 1B1-2) after 3 d (i.p., 1 mg in 200 µL PBS), 4 d (i.n., 0.15 mg in 30 µL PBS) and 6 d.p.i (i.p., 1 mg in 200 µL PBS) [27]. rIL-27 (200 ng in 100 µL PBS; eBioscience) was injected i.p. from 1–7 d.p.i. (early) or 5–9/10 d.p.i. (late treatment). In parallel, mice were injected solely with PBS (NT) as control. NK cell depletion was performed by i.p injection of anti-NK1.1 depleting antibody (500 µg in 500 µL; clone PK136) at −1, 1 and 5 d.p.i.

### Histolopathological analysis

Lung samples were fixed with 4% formaldehyde, embedded in paraffin and stained with hematoxylin and eosin (H&E). Images were acquired using an AxioImager Z1 microscope equipped with a charge-coupled device (CCD) camera (AxioCam MRm) and processed with AxioVision software (all purchased from Carl Zeiss MicroImaging, Inc.). H&E stained lung sections were scored in a blinded manner as follows: (0) normal, (1) minor perivascular inflammation around large blood vessels, (2) moderate perivascular and peribronchial inflammation, (3) increased perivascular and peribronchial inflammation, (4) severe formation of perivascular, peribronchial, and interstitial inflammation.

### Antibodies and flow cytometry analysis

The following murine monoclonal antibodies (mAbs) were purchased from BD Biosciences or eBioscience: CD4 (RM4-5), Ly6-G (RB6-8C5), CD11c (N418), CD49b (pan-NK), CD11b (M1-70), CD8a^+^ (53-6.7), CD62L (16A/MEL-14), CD3 (145-2C11), IFN-γ (AN18.17.24), IL-10 (JES5-A6E3), IL-17A (TC11-18H10), TNF-α (MP6-XT22), CD107 (1D4B). CD31 (MEC13.3), ICAM-1 (KAT1) and MHCII (M5/114) were obtained in house. In certain cases, PI was added at 40 µg/ml to the cells immediately prior to cell acquisition.

### Statistical analysis

Data are means ± s.d. or s.e.m. Statistical tests used include Kaplan-Meier log-rank survival test, and unpaired two-tailed Students *t* test. All *P* values>0.05 are considered not to be significant.

## Supporting Information

Figure S1 **Similar levels of IFNα in BAL but increased numbers of TNF-α^+^CD4^+^ T cells in the lungs of influenza virus infected** ***Il-27ra^−/−^*** **mice.** Levels of IFNα in BAL (A), and numbers of TNF-α and IL-4-producing CD4^+^ T cells in the infected lungs of *Il-27ra^−/−^* mice at 9 d.p.i. (B, C).
(PDF)

Figure S2 **IL-10 production by CD4^+^ T cells during influenza requires IL-27.** Numbers of IL-10^+^IFN-γ^+^ double-positive (A), IL-10^+^ single-positive (C) CD4^+^ T cells and ratio IL-10^+^/IFN-γ^+^ (B) in the infected respiratory tract of *Il-27ra^−/−^* mice at 9 d.p.i.
(PDF)

Figure S3 **Induction of IL-10 in CD4^+^ T cells partially requires STAT4 but not IL-12.** Frequencies and numbers of IL-10^+^IFN-γ^+^CD4^+^ T cells in the lungs of influenza virus infected *Stat4^−/−^* (A) or *Il-12p40^−/−^* (B) mice. Viral load in *Stat4^−/−^* mice (C).
(PDF)

Figure S4 **IL-10 deficiency results in increased frequencies of IL-17 but not IFN-γ^+^ T cells in the lungs.** Antigen-specific IFN-γ or IL-17-producing CD8^+^ T cells in the BAL and lungs of infected *Il-10^−/−^* mice at 7 d.p.i.
(PDF)

Figure S5 **Blocked IL-10 signaling during influenza impaired IL-17, but not IFN-γ expression in T cells.** The numbers of virus-specific IL-17 or IFN-γ-producing CD4+ (A) or CD8+ (B) T cells in the lungs of infected WT mice were assessed by FACS after blocking IL-10 signaling using an anti-IL-10 receptor blocking-antibody (αIL-10R). Viral load in *IL-10^−/−^* mice (C).
(PDF)

Figure S6 **IL-27 suppresses IFN-γ production by CD8^+^ T cells independently from IL-10 without inducing activation-induced cell death (AICD).** (A) IL-27-dependent reduction of IFN-γ production by Tc1 cells after 3 and 4 days in culture. (B) CFSE staining of Tc1 cells in the presence of rIL-27.
(PDF)

Figure S7 **Deficiency in IL-10 does not aggravate disease course in influenza virus infected mice.** Weight loss comparison of *Il-10^−/−^* versus WT mice after sublethal influenza virus infection.
(PDF)

Figure S8 **rIL-27 treatment during the later phase of influenza does not affect antigen-specific CD4^+^ T cell cytokine responses, CD8^+^ (CTL) activity or levels of virus-specific antibody.** The numbers of IL-17, IFN-γ or TNF-α positive CD4+ T cells (A), of CD107 or IFNγ positive CD8+ T cells (B) in lungs and/or BAL upon peptide restimulation as well as virus-specific antibody titers (C) in serum of infected mice after late rIL-27 treatment.
(PDF)

Figure S9 **Minimal role of NK cells in viral clearance during influenza.** Depletion of NK-cells (A) and its effects on viral titers (B) in WT mice at 7 d.p.i.
(PDF)

Figure S10 **IL-27 suppresses IL-1 or IL-6-induced chemokine production in lung endothelial cells.** Levels of IL-1 or IL-6 produced by sorted lung endothelial cells after rIL-27 treatment *in vitro*.
(PDF)

Figure S11 **Minimal levels of IL-17 were found in zymosan-induced peritonitis.** IL-17 levels in the peritoneal lavage of mice 24 h post injection with zymosan was measured by ELISA.
(PDF)

Figure S12 **Decreased neutrophil, but not NK cell accumulation after late IL-27 treatment is dependent on IL-10 and IL-10 is required for reduction of chemokine production.** (A) Frequencies of neutrophils and NK cells of infected *Il-10^−/−^* mice treated with rIL-27. (B) Frequencies of neutrophils and NK cells of infected WT mice after anti-IL-10R antibody + rIL-27 treatment. (C) Chemokine levels in the BAL fluid of *Il-10^−/−^* or WT mice infected with influenza virus and treated with rIL-27.
(PDF)

Figure S13 **Level of IL-27 in lung homogenates or plasma of mice after late IL-27 treatment.**
(PDF)

Figure S14 **Control of inflammation by local and systemic action of IL-27 in influenza.** A cartoon depicting the hypothetical scheme of interactions after rIL-27 treatment.
(PDF)

Table S1 **Influenza virus peptides used for T cell restimulation** ***in vitro*** **.**
(PDF)

Table S2 **Primers were used for qRT-PCR analysis.**
(PDF)
