# Supplementary material for: Timed Action of IL-27 Protects from Immunopathology while Preserving Defense in Influenza
Source: PLoS Pathog. 2014 May 8;10(5):e1004110. doi: 10.1371/journal.ppat.1004110 (PMC4014457; doi:10.1371/journal.ppat.1004110)
Supplement: Figure S1 — Similar levels of IFNα in BAL but increased numbers of TNF-α+CD4+ T cells in the lungs of influenza virus infected Il-27ra−/− mice. Levels of IFNα in BAL (A), and numbers of TNF-α and IL-4-producing CD4+ T cells in the infected lungs of Il-27ra−/− mice at 9 d.p.i. (B, C). (PDF) [file ppat.1004110.s001.pdf]

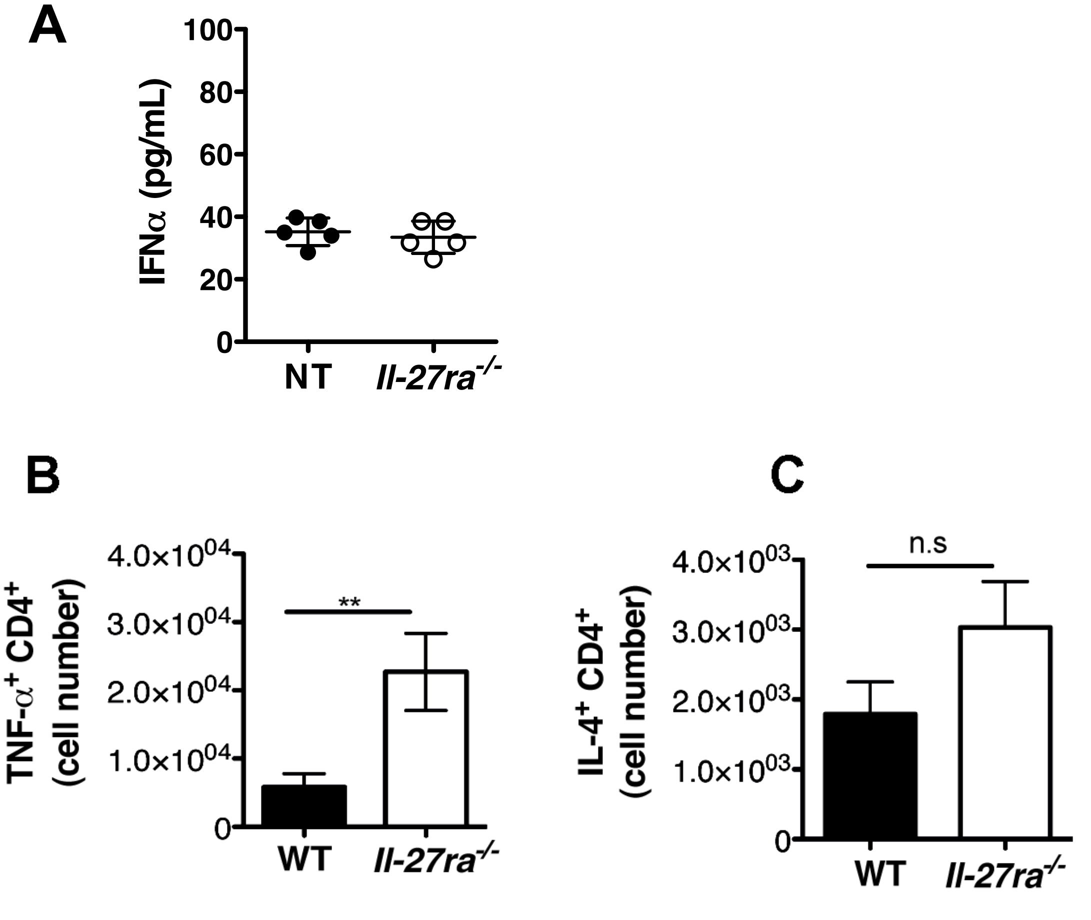

Supplementary Figure 1. Similar levels of IFNα in BAL but increased numbers of TNF-α^{+}CD4^{+} T cells in the lungs of influenza virus infected *Il-27ra$^{-/-}$* mice. *Il-27ra$^{-/-}$* (*n* = 4) or C57BL/6 (*n* = 4) mice were infected with 2500 EID influenza virus. At 9 d.p.i, levels of IFNα were assayed with bead array in BAL **(A),** and cells isolated from lung tissue were analyzed by FACS to determine the numbers of TNF-α or IL-4-producing CD4^{+} T cells **(B, C)** after *in vitro* restimulation with PMA/ionomycin. *P* values were determined by unpaired two-tailed Student's *t* test. Values are means ± s.d.; n.s, not significant.
