# Supplementary material for: Timed Action of IL-27 Protects from Immunopathology while Preserving Defense in Influenza
Source: PLoS Pathog. 2014 May 8;10(5):e1004110. doi: 10.1371/journal.ppat.1004110 (PMC4014457; doi:10.1371/journal.ppat.1004110)
Supplement: Figure S2 — IL-10 production by CD4+ T cells during influenza requires IL-27. Numbers of IL-10+IFN-γ+ double-positive (A), IL-10+ single-positive (C) CD4+ T cells and ratio IL-10+/IFN-γ+ (B) in the infected respiratory tract of Il-27ra−/− mice at 9 d.p.i. (PDF) [file ppat.1004110.s002.pdf]

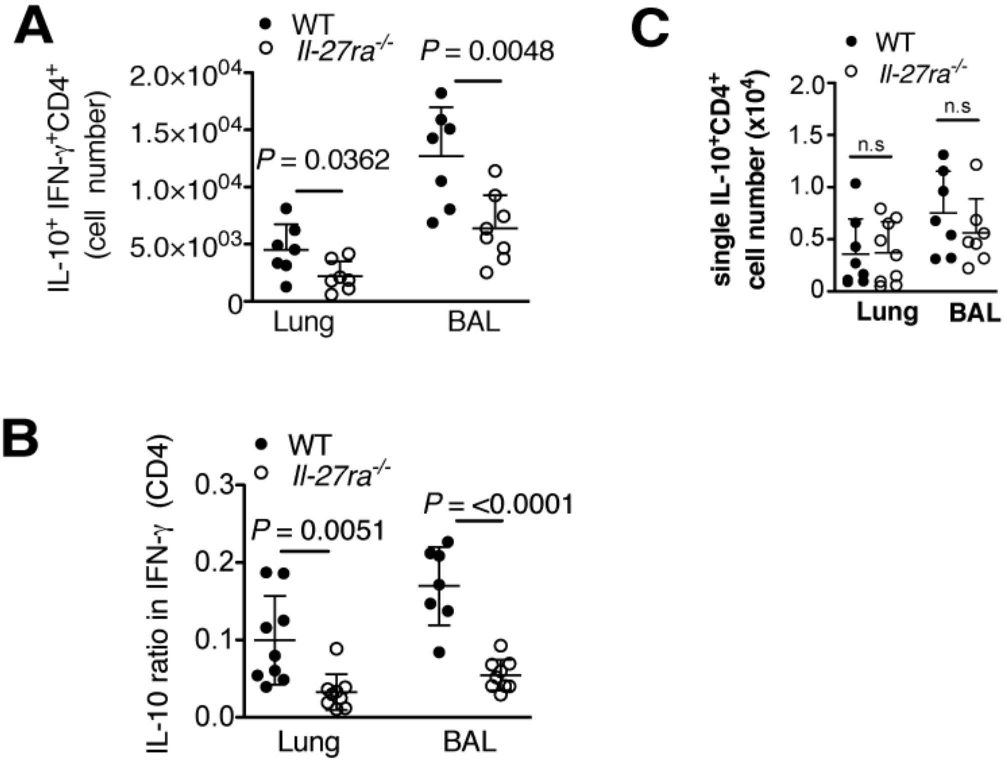

Supplementary Figure 2. IL-10 production by $CD4^+$ T cells during influenza requires IL-27. *Il-27ra*$^{-/-}$, or wild-type (WT) C57BL/6 mice were infected with 2500 EID influenza virus. Numbers of **(A)** IL-10^+IFN-$\gamma^+$$CD4^+$ or **(C)** single IL-$10^+$$CD4^+$ T cells in the BAL and lungs of *Il-27ra*$^{-/-}$ mice were analyzed by FACS after *in vitro* restimulation with PMA/iono. **(B)** Ratios of IL-10:IFN-γ in gated $CD4^+$ T cells from infected *Il-27ra*$^{-/-}$ mice. All data sets were analyzed at 9 d.p.i and are pooled from two independent experiments. *P* values were determined by unpaired two-tailed Student's *t* test. Values are means ± s.d.; ns, not significant.
