# Supplementary material for: Timed Action of IL-27 Protects from Immunopathology while Preserving Defense in Influenza
Source: PLoS Pathog. 2014 May 8;10(5):e1004110. doi: 10.1371/journal.ppat.1004110 (PMC4014457; doi:10.1371/journal.ppat.1004110)
Supplement: Figure S3 — Induction of IL-10 in CD4+ T cells partially requires STAT4 but not IL-12. Frequencies and numbers of IL-10+IFN-γ+CD4+ T cells in the lungs of influenza virus infected Stat4−/− (A) or Il-12p40−/− (B) mice. Viral load in Stat4−/− mice (C). (PDF) [file ppat.1004110.s003.pdf]

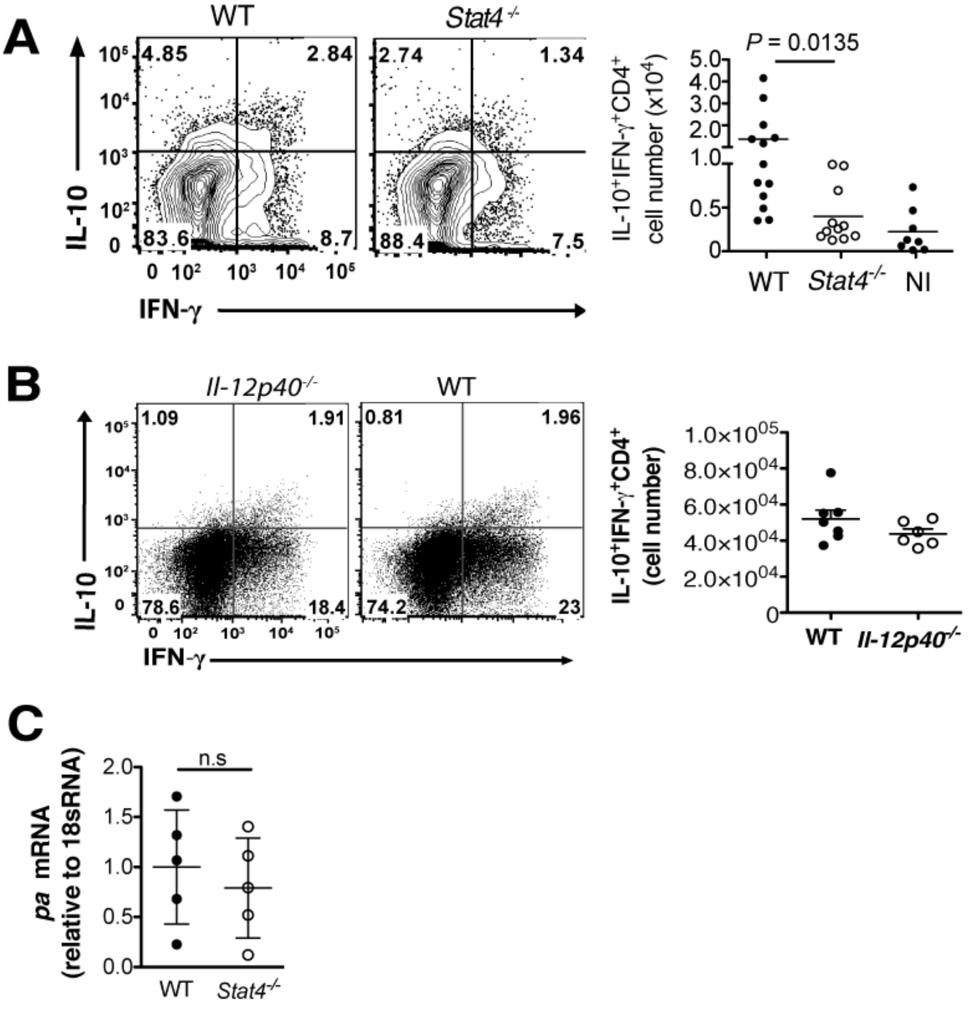

Supplementary Figure 3. Induction of IL-10 in CD4$^+$ T cells partially requires STAT4 but not IL-12. (A) *Stat4$^{-/-}$*, **(B)** *Il-27p40$^{-/-}$* or WT mice were infected with influenza virus. Lungs were analyzed for IL-10$^+$IFN-γ$^+$CD4$^+$ T cells by FACS and **(C)** viral polymerase (*pa*) mRNA expression by qRT-PCR. FACS plots represent data from two independent experiments with similar results. All data sets were analzed at 9 d.p.i and are pooled from two independent experiments. *P* values were determined by unpaired two-tailed Student's *t* test. Values are means ± s.d.; ns, not significant.
