# Supplementary material for: Timed Action of IL-27 Protects from Immunopathology while Preserving Defense in Influenza
Source: PLoS Pathog. 2014 May 8;10(5):e1004110. doi: 10.1371/journal.ppat.1004110 (PMC4014457; doi:10.1371/journal.ppat.1004110)
Supplement: Figure S4 — IL-10 deficiency results in increased frequencies of IL-17 but not IFN-γ+ T cells in the lungs. Antigen-specific IFN-γ or IL-17-producing CD8+ T cells in the BAL and lungs of infected Il-10−/− mice at 7 d.p.i. (PDF) [file ppat.1004110.s004.pdf]

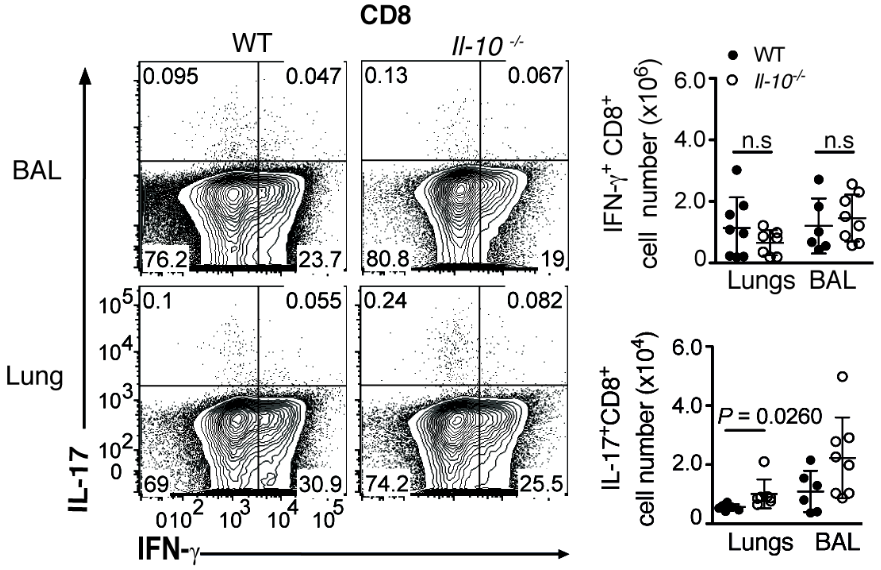

Supplementary Figure 4. IL-10 deficiency results in increased frequencies of IL-17 but not IFN-γ^{+} T cells in the lungs. *Il-10$^{-/-}$* or C57BL/6 (WT) mice were infected with a sublethal dose influenza virus. At 7 d.p.i., antigen-specific IFN-γ or IL-17-producing CD8^{+} T cells in the BAL and lungs were assessed by FACS. Data were pooled from two independent experiments with similar results. *P* values were determined by unpaired two-tailed Student's *t* test. Values are means ± s.d.; ns, not significant.
