# Supplementary material for: Timed Action of IL-27 Protects from Immunopathology while Preserving Defense in Influenza
Source: PLoS Pathog. 2014 May 8;10(5):e1004110. doi: 10.1371/journal.ppat.1004110 (PMC4014457; doi:10.1371/journal.ppat.1004110)
Supplement: Figure S5 — Blocked IL-10 signaling during influenza impaired IL-17, but not IFN-γ expression in T cells. The numbers of virus-specific IL-17 or IFN-γ-producing CD4+ (A) or CD8+ (B) T cells in the lungs of infected WT mice were assessed by FACS after blocking IL-10 signaling using an anti-IL-10 receptor blocking-antibody (αIL-10R). Viral load in IL-10−/− mice (C). (PDF) [file ppat.1004110.s005.pdf]

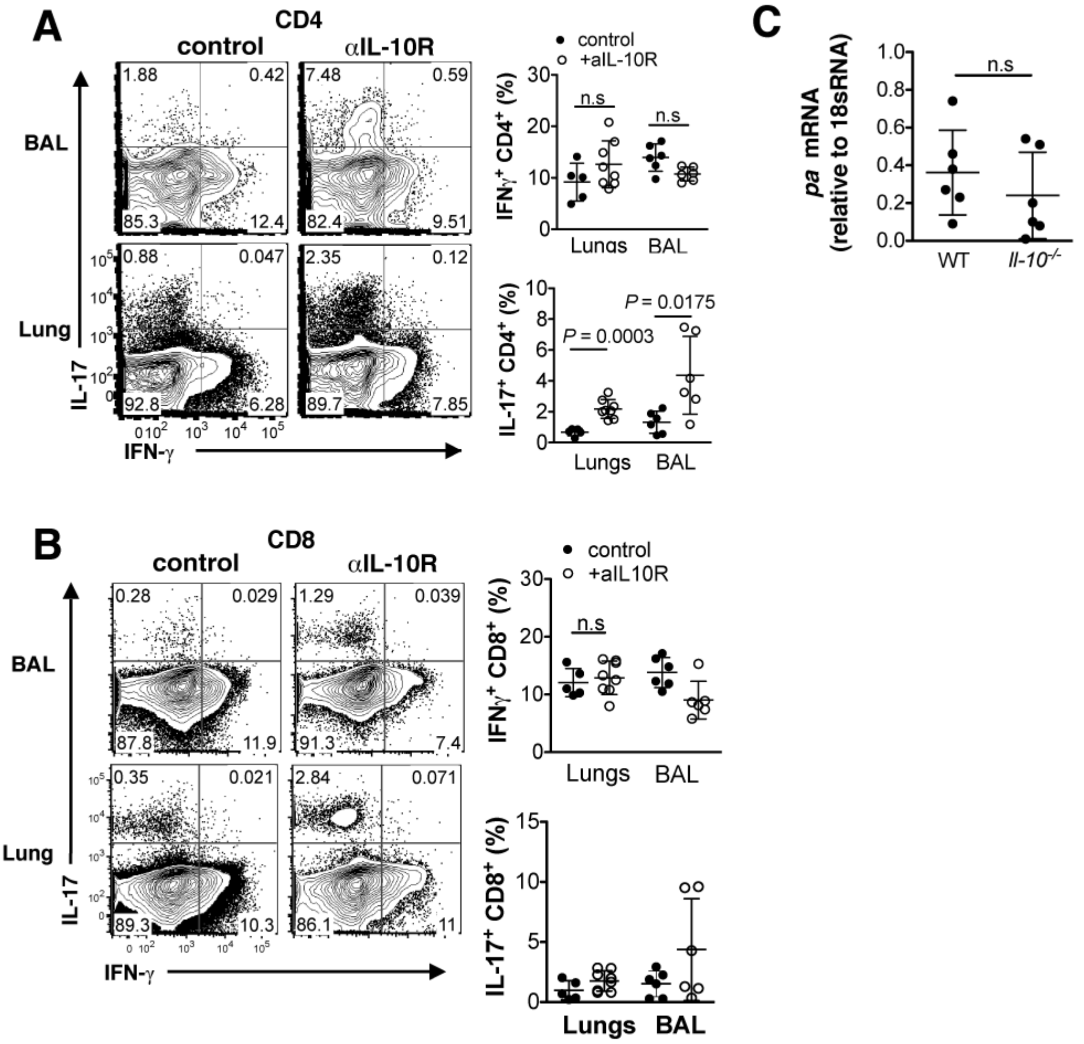

Supplementary Figure 5. Blocked IL-10 signaling during influenza impaired IL-17, but not IFN-γ expression in T cells. C57BL/6 mice were infected with a sublethal dose influenza virus then administered with anti-IL-10 receptor blocking-antibody (αIL-10R). influenza peptide-specific cytokine production by **(A)** $CD4^{+}$ or **(B)** $CD8^{+}$ T cells was detected through FACS after *in vitro* restimulation with immunodominant influenza virus peptides. **(C)** viral (*pa*) polymerase mRNA expression of sublethally infected *Il-10*$^{-/-}$ or C57BL/6 mice at 7 d.p.i. was measured by qRT-PCR. FACS plots represent data from two independent experiments. Remaining data sets are pooled from two independent experiments. *P* values were determined by unpaired two-tailed Student's *t* test. Values are means ± s.d.; ns, not significant.
