# Supplementary material for: Timed Action of IL-27 Protects from Immunopathology while Preserving Defense in Influenza
Source: PLoS Pathog. 2014 May 8;10(5):e1004110. doi: 10.1371/journal.ppat.1004110 (PMC4014457; doi:10.1371/journal.ppat.1004110)
Supplement: Figure S6 — IL-27 suppresses IFN-γ production by CD8+ T cells independently from IL-10 without inducing activation-induced cell death (AICD). (A) IL-27-dependent reduction of IFN-γ production by Tc1 cells after 3 and 4 days in culture. (B) CFSE staining of Tc1 cells in the presence of rIL-27. (PDF) [file ppat.1004110.s006.pdf]

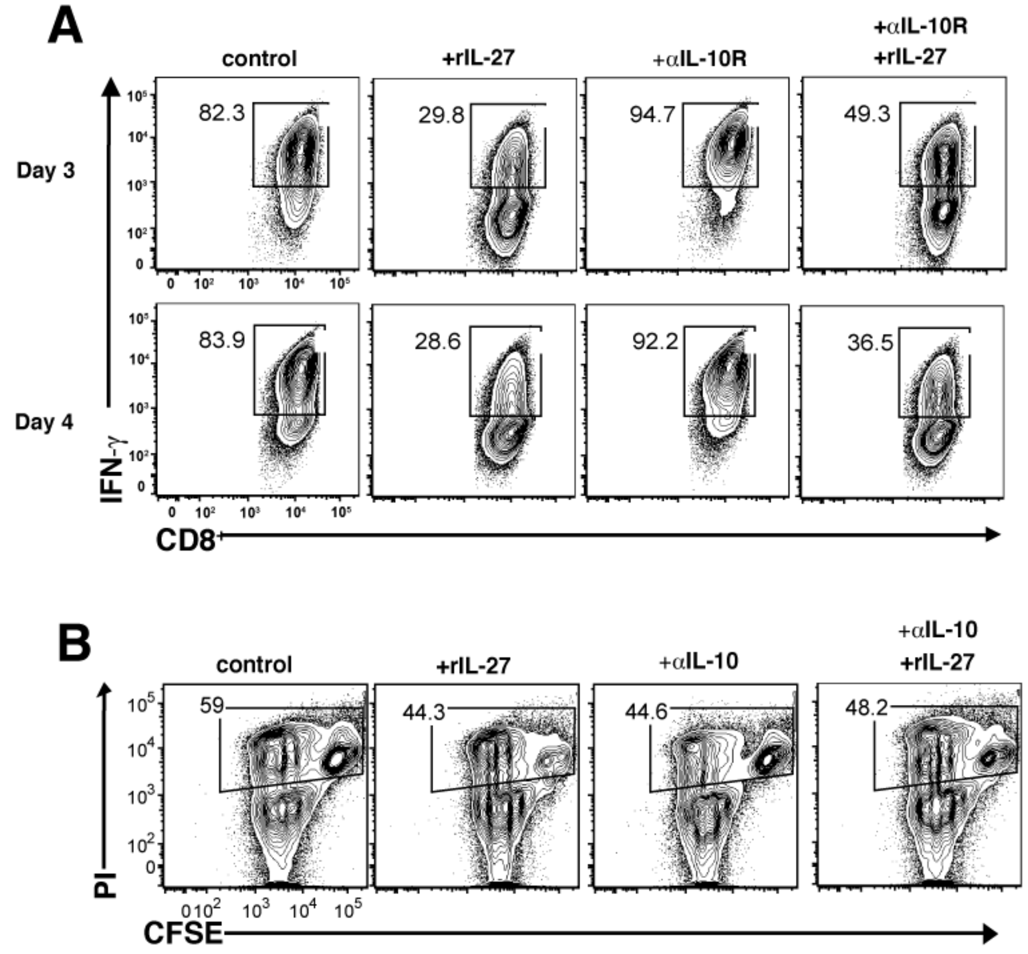

Supplementary Figure 6. IL-27 suppresses IFN-γ production by CD8$^+$ T cells independently from IL-10 without inducing activation-induced cell death (AICD). **(A)** Naive CD8$^+$ T cells were activated by plate-bound anti-CD3 and anti-CD28 in Tc1 polarizing conditions and were supplemented with rIL-27 and/or αIL-10R antibody. After 3d in culture, cells were transferred to plates without stimuli then media was added with rIL-2 and/or rIL-27 and αIL-10R antibody for an additional 2 days for a total of 5 days in culture. FACS was performed to detect IFN-γ-producing Tc1 cells in various conditions. **(B)** Previously CFSE-labelled Tc1 cells were cultured as mentioned above then stained with propidium iodide (PI) prior to FACS acquisition to determine cell death. All data sets represent data obtained from at least two independent experiments with similar results.
