# Supplementary material for: Timed Action of IL-27 Protects from Immunopathology while Preserving Defense in Influenza
Source: PLoS Pathog. 2014 May 8;10(5):e1004110. doi: 10.1371/journal.ppat.1004110 (PMC4014457; doi:10.1371/journal.ppat.1004110)
Supplement: Figure S7 — Deficiency in IL-10 does not aggravate disease course in influenza virus infected mice. Weight loss comparison of Il-10−/− versus WT mice after sublethal influenza virus infection. (PDF) [file ppat.1004110.s007.pdf]

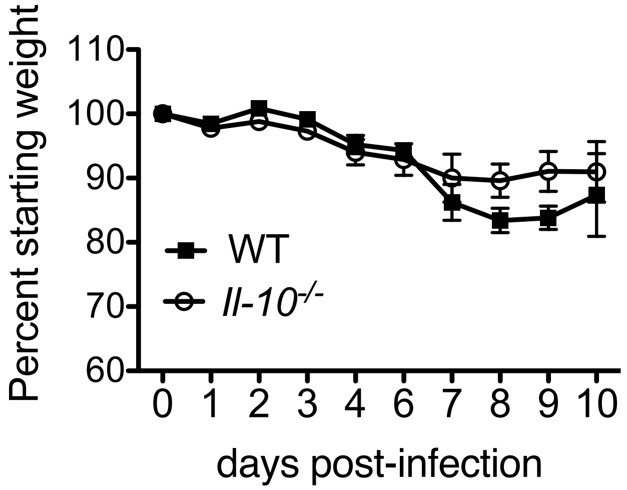

Supplementary Figure 7. Deficiency in IL-10 does not aggravate disease course in influenza virus infected mice. *Il-10*$^{-/-}$ and C57BL/6 mice were infected with a sublethal dose of influenza virus and weight loss was monitored daily. Values are means ± s.d.
