# Supplementary material for: Timed Action of IL-27 Protects from Immunopathology while Preserving Defense in Influenza
Source: PLoS Pathog. 2014 May 8;10(5):e1004110. doi: 10.1371/journal.ppat.1004110 (PMC4014457; doi:10.1371/journal.ppat.1004110)
Supplement: Figure S8 — rIL-27 treatment during the later phase of influenza does not affect antigen-specific CD4+ T cell cytokine responses, CD8+ (CTL) activity or levels of virus-specific antibody. The numbers of IL-17, IFN-γ or TNF-α positive CD4+ T cells (A), of CD107 or IFNγ positive CD8+ T cells (B) in lungs and/or BAL upon peptide restimulation as well as virus-specific antibody titers (C) in serum of infected mice after late rIL-27 treatment. (PDF) [file ppat.1004110.s008.pdf]

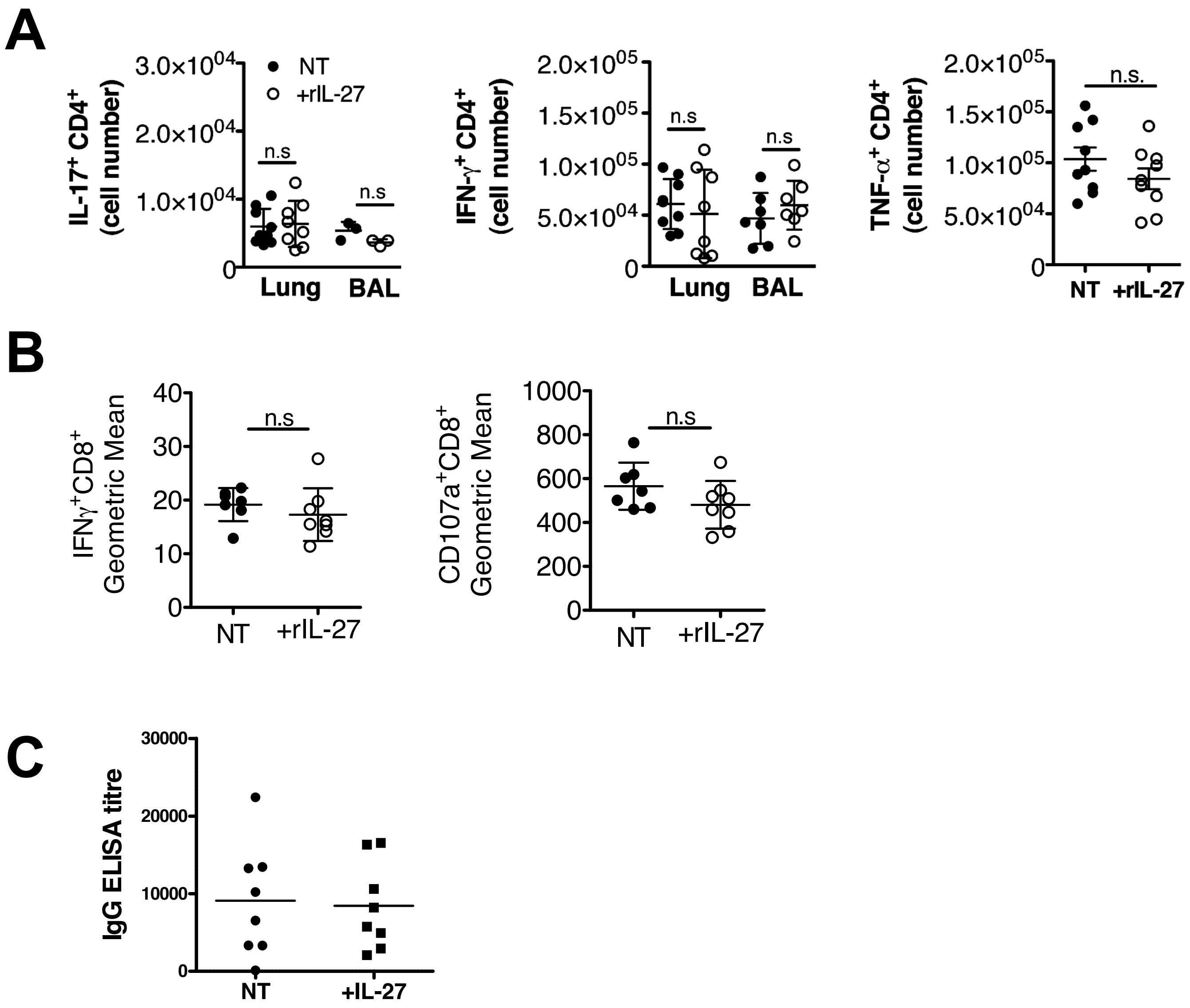

Supplementary Figure 8. rIL-27 treatment during the later phase of influenza does not affect antigen-specific $CD4^+$ T cell cytokine responses, $CD8^+$ (CTL) activity or levels of virus-specific antibody. C57BL/6 mice were infected with influenza virus and treated with rIL-27 daily from 5-9 d.p.i. At 9 d.p.i., (A) influenza peptide-specific cytokine production by $CD4^+$ T cells from lung and BAL, and (B) IFNγ production or CD107 surface expression by CD8+ cells from lung were analyzed by FACS after restimulation with influenza virus peptides. (C) Virus-specific antibody titers were analyzed with ELISA in plates coated with PR/8/34 virus. *P* values were determined by unpaired two-tailed Student's *t* test. Values are means ± s.d.; n.s, not significant

.
