# Supplementary material for: Timed Action of IL-27 Protects from Immunopathology while Preserving Defense in Influenza
Source: PLoS Pathog. 2014 May 8;10(5):e1004110. doi: 10.1371/journal.ppat.1004110 (PMC4014457; doi:10.1371/journal.ppat.1004110)
Supplement: Figure S9 — Minimal role of NK cells in viral clearance during influenza. Depletion of NK-cells (A) and its effects on viral titers (B) in WT mice at 7 d.p.i. (PDF) [file ppat.1004110.s009.pdf]

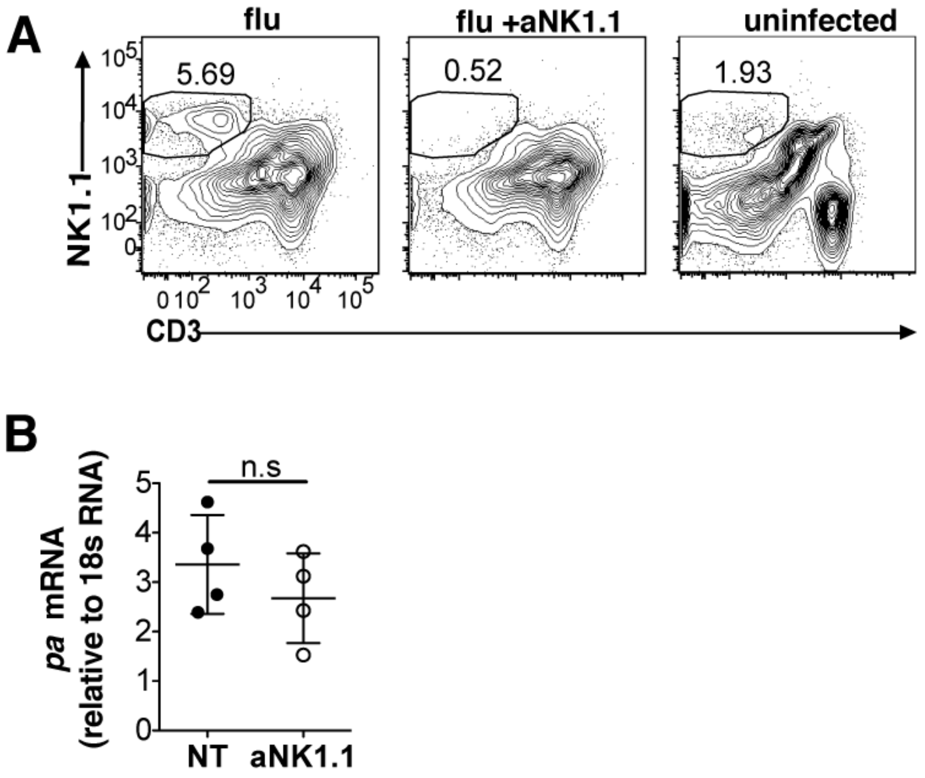

Supplementary Figure 9. Minimal role of NK cells in viral clearance during influenza. Depletion of NK cells from influenza virus infected mice was performed by administration of a depleting anti-NK1.1 monoclonal antibody at -1, 1 and 5 d.p.i. **(A)** NK cell depletion was confirmed by FACS. **(B)** Viral *pa* mRNA from NK cell-depleted mice was measured by qRT-PCR at day 7 p.i. *P* values were determined by unpaired two-tailed Student's *t* test. Values are means ± s.d.; n.s, not significant.
