# Supplementary material for: Timed Action of IL-27 Protects from Immunopathology while Preserving Defense in Influenza
Source: PLoS Pathog. 2014 May 8;10(5):e1004110. doi: 10.1371/journal.ppat.1004110 (PMC4014457; doi:10.1371/journal.ppat.1004110)
Supplement: Figure S10 — IL-27 suppresses IL-1 or IL-6-induced chemokine production in lung endothelial cells. Levels of IL-1 or IL-6 produced by sorted lung endothelial cells after rIL-27 treatment in vitro. (PDF) [file ppat.1004110.s010.pdf]

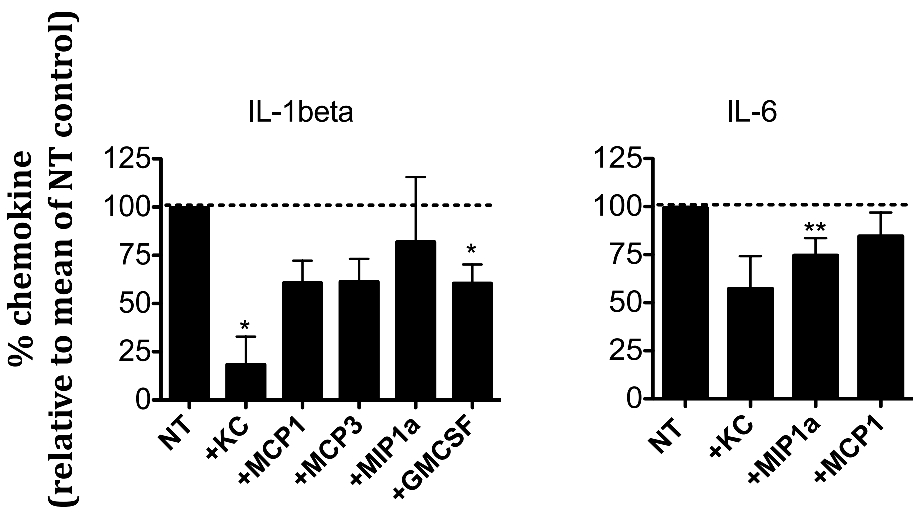

Supplementary Figure 10. IL-27 suppresses IL-1 or IL-6-induced chemokine production in lung endothelial cells. Sorted lung endothelial cells ($CD45^{-}$ $CD31^{+}$) from C57BL/6 mice were stimulated with IL-1 or IL-6 in the presence of absence of IL-27. After 24h, levels of various chemokines were measured.
