# Supplementary material for: Timed Action of IL-27 Protects from Immunopathology while Preserving Defense in Influenza
Source: PLoS Pathog. 2014 May 8;10(5):e1004110. doi: 10.1371/journal.ppat.1004110 (PMC4014457; doi:10.1371/journal.ppat.1004110)
Supplement: Figure S11 — Minimal levels of IL-17 were found in zymosan-induced peritonitis. IL-17 levels in the peritoneal lavage of mice 24 h post injection with zymosan was measured by ELISA. (PDF) [file ppat.1004110.s011.pdf]

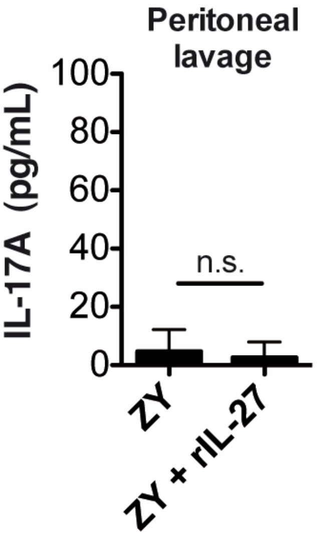

Supplementary Figure 11. Minimal levels of IL-17 were found in zymosan-induced peritonitis (compare to Fig. 4F). Zymosan (ZY) alone or in combination with rIL-27 (ZY+ rIL-27) were injected into C57BL/6 intraperitoneally. 24h post-injection, peritoneal lavage was obtained and analyzed for IL-17 by ELISA. Bars represent n = 7 (ZY) and n = 9 (ZY + rIL-27). Detection level: 4 pg according to manufacturer. P values were determined by unpaired two-tailed Student's t test. Values are means ± s.d.; n.s, not significant.
