# Supplementary material for: Timed Action of IL-27 Protects from Immunopathology while Preserving Defense in Influenza
Source: PLoS Pathog. 2014 May 8;10(5):e1004110. doi: 10.1371/journal.ppat.1004110 (PMC4014457; doi:10.1371/journal.ppat.1004110)
Supplement: Figure S12 — Decreased neutrophil, but not NK cell accumulation after late IL-27 treatment is dependent on IL-10 and IL-10 is required for reduction of chemokine production. (A) Frequencies of neutrophils and NK cells of infected Il-10−/− mice treated with rIL-27. (B) Frequencies of neutrophils and NK cells of infected WT mice after anti-IL-10R antibody + rIL-27 treatment. (C) Chemokine levels in the BAL fluid of Il-10−/− or WT mice infected with influenza virus and treated with rIL-27. (PDF) [file ppat.1004110.s012.pdf]

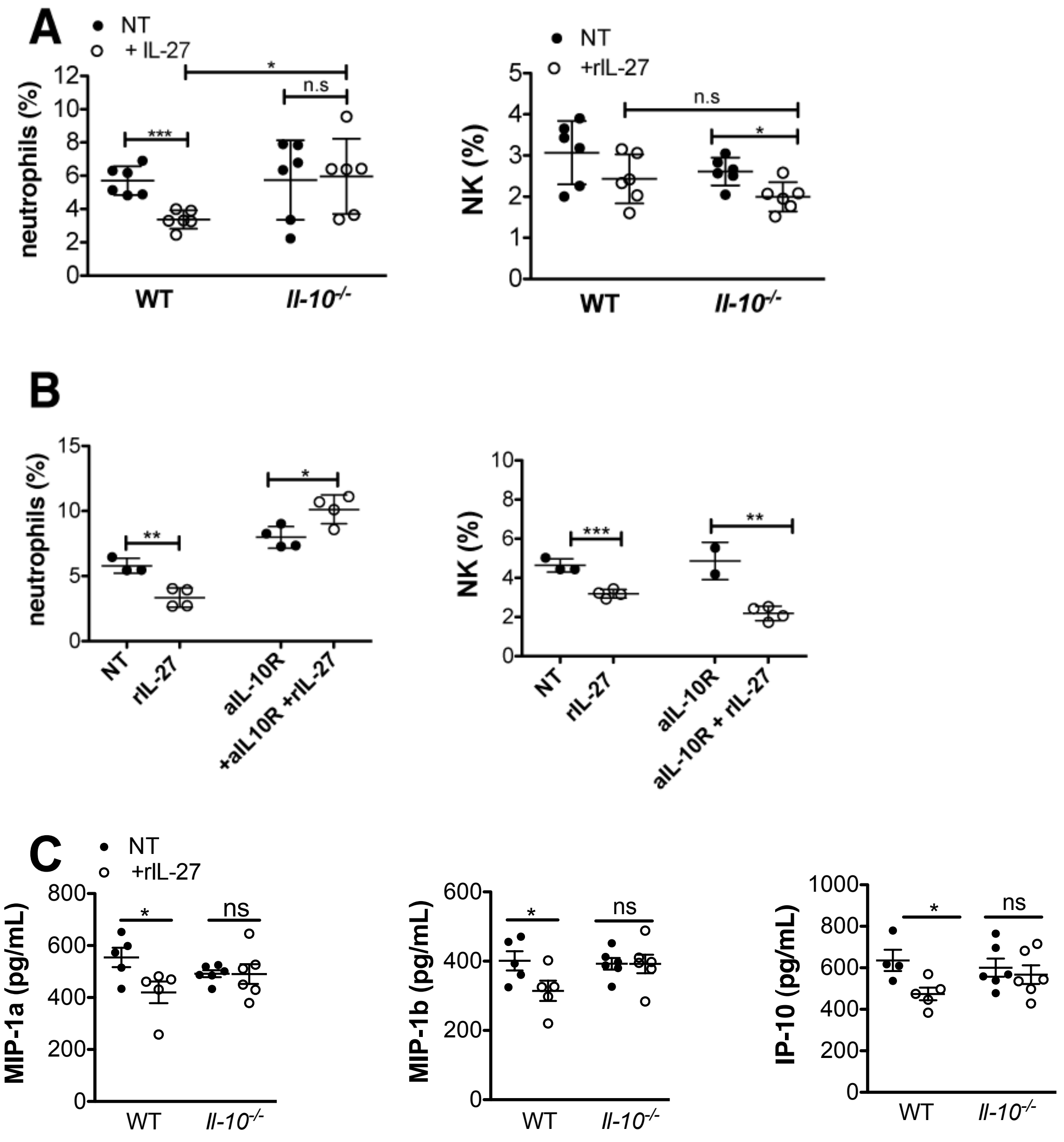

Supplementary Figure 12. Decreased neutrophil, but not NK cell accumulation after late IL-27 treatment is dependent on IL-10 and IL-10 is required for reduction of chemokine production. (A) *Il-10*$^{-/-}$ or C57BL/6 mice were infected with influenza virus and treated with rIL-27 and daily from day 1-6 p.i. At 7 d.p.i, neutrophil or NK cell frequencies were determined by FACS. **(B)** Influenza virus infected C57BL/6 mice were simultaneously treated with rIL-27 and anti-IL-10R antibody (aIL-10R) daily from 1-6 d.p.i. At 7 d.p.i, neutrophil or NK cell frequencies were determined by FACS. (**C**) Chemokine levels in the BAL fluid of *Il-10*$^{-/-}$ or C57BL/6 mice infected with influenza virus and treated with rIL-27. *P* values were determined by unpaired two-tailed Student's *t* test. Values are means ± s.d. **P*<0.05; ***P*<0.01; ****P*<0.0001; ns, not significant.
