# Supplementary material for: Timed Action of IL-27 Protects from Immunopathology while Preserving Defense in Influenza
Source: PLoS Pathog. 2014 May 8;10(5):e1004110. doi: 10.1371/journal.ppat.1004110 (PMC4014457; doi:10.1371/journal.ppat.1004110)
Supplement: Figure S13 — Level of IL-27 in lung homogenates or plasma of mice after late IL-27 treatment. (PDF) [file ppat.1004110.s013.pdf]

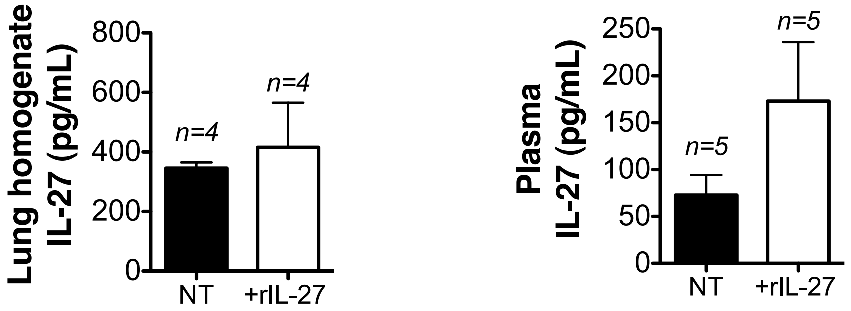


**Supplementary Figure 13. Level of IL-27 in lung homogenates or plasma of mice after late IL-27 treatment.** C57BL/6 mice were challenged influenza virus then treated daily with rIL-27 from 5-9 d.p.i. At 9 d.p.i, homogenates of lung tissues were obtained by mashing the lungs (approximately 200 mg) with 10 mL of medium. Levels of IL-27 in the lung homogenates or plasma were determined by ELISA.
