# Supplementary material for: Timed Action of IL-27 Protects from Immunopathology while Preserving Defense in Influenza
Source: PLoS Pathog. 2014 May 8;10(5):e1004110. doi: 10.1371/journal.ppat.1004110 (PMC4014457; doi:10.1371/journal.ppat.1004110)
Supplement: Figure S14 — Control of inflammation by local and systemic action of IL-27 in influenza. A cartoon depicting the hypothetical scheme of interactions after rIL-27 treatment. (PDF) [file ppat.1004110.s014.pdf]

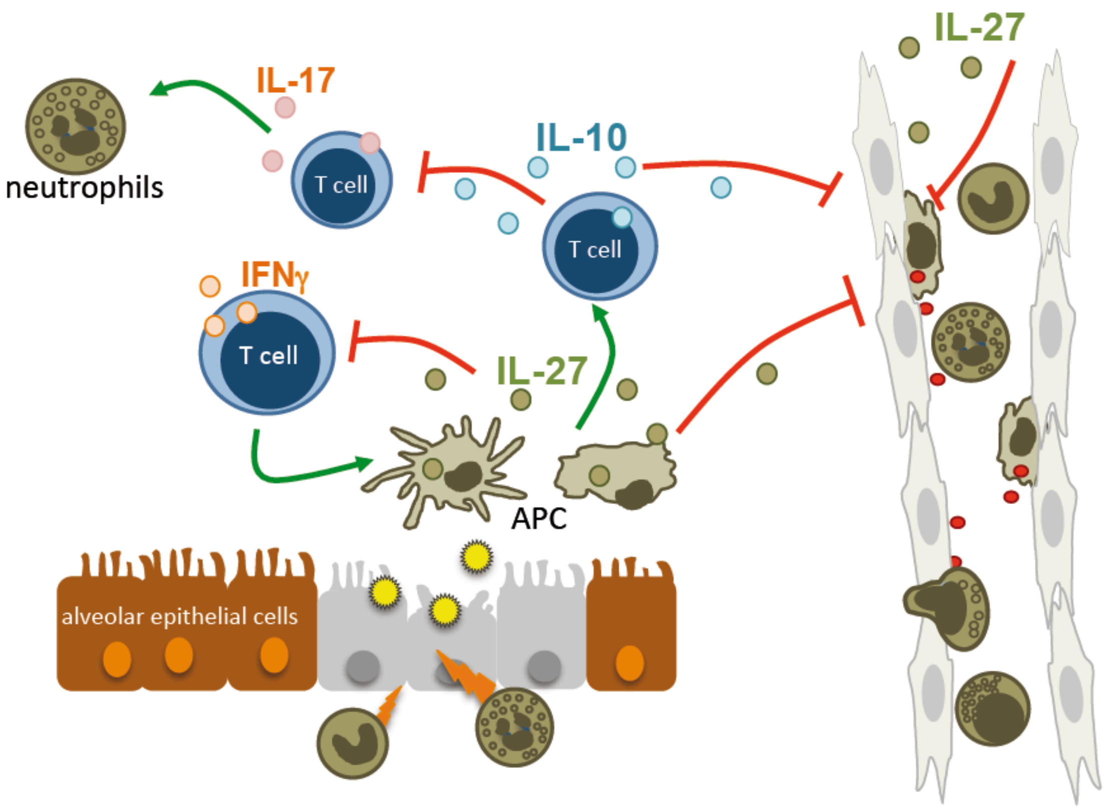

Supplementary Figure 14. Control of inflammation by local and systemic action of IL-27 in influenza. Hypothetical scheme of interactions. Systemic administration of rIL-27 during influenza alleviates immunopathology by reducing neutrophil and monocyte influx to the lungs and predominantly might act by affecting chemokine (red dots) production and deposition intravascularly.
