# Supplementary material for: Timed Action of IL-27 Protects from Immunopathology while Preserving Defense in Influenza
Source: PLoS Pathog. 2014 May 8;10(5):e1004110. doi: 10.1371/journal.ppat.1004110 (PMC4014457; doi:10.1371/journal.ppat.1004110)
Supplement: Table S1 — Influenza virus peptides used for T cell restimulation in vitro . (PDF) [file ppat.1004110.s015.pdf]

Supplementary Table 1. Influenza virus peptides used for T cell restimulation *in vitro*.

CD8$^+$ T cell	
nucleoprotein $(NP)_{366-374}$	ASNEMNDAM
acid polymerase A $(PA)_{224-233}$	SSLENFRAYV
nonstructural protein 2 $(NS2)_{114-121}$	RTFSFQLI
CD4$^+$ T cell	
nucleoprotein $(NP)_{261-275}$	RSALILRGSVAHKSC
hemagglutinin $(HA)_{126-138}$	HNTNGVTAACHSE
