# Supplementary material for: Timed Action of IL-27 Protects from Immunopathology while Preserving Defense in Influenza
Source: PLoS Pathog. 2014 May 8;10(5):e1004110. doi: 10.1371/journal.ppat.1004110 (PMC4014457; doi:10.1371/journal.ppat.1004110)
Supplement: Table S2 — Primers were used for qRT-PCR analysis. (PDF) [file ppat.1004110.s016.pdf]

Supplementary Table 2. Primers were used for qRT-PCR analysis.

HPRT forward	ATCATTATGCCGAGGATTTGGAA
HPRT reverse	TTGAGCACACACAGAGGGCC
18s forward	GATCCATTGGAGGGCAAGTCT
18s reverse	GCAGCAACTTTAATATACGCTATTGC
EBI3 forward	GGCTGAGCGAATCATCAAG
EBI3 reverse	CTGTGAGGTCCTGAGCTGAC
IL-27p28 forward	CTGGTACAAGCTGGTTCCTG
IL-27p28reverse	TCAGAGTCAGAGAGGTGATGC
IL-10 forward	GCACTACCAAAGCCACAAG
IL-10 reverse	TGTTTGAAAGAAAGTCTTCACCTG
PA forward	CGGTCCAAATTCCTGCTGA
PA reverse	CATTGGGTTCCTTCCATCCA
